# Handling Uncertainty in Dynamic Models: The Pentose Phosphate Pathway in *Trypanosoma brucei*

**DOI:** 10.1371/journal.pcbi.1003371

**Published:** 2013-12-05

**Authors:** Eduard J. Kerkhoven, Fiona Achcar, Vincent P. Alibu, Richard J. Burchmore, Ian H. Gilbert, Maciej Trybiło, Nicole N. Driessen, David Gilbert, Rainer Breitling, Barbara M. Bakker, Michael P. Barrett

**Affiliations:** 1 Wellcome Trust Centre for Molecular Parasitology, Institute of Infection, Immunity and Inflammation and Glasgow Polyomics, College of Medical, Veterinary and Life Sciences, University of Glasgow, Glasgow, United Kingdom; 2 Systems and Synthetic Biology Group, Department of Chemical and Biological Engineering, Chalmers University of Technology, Gothenburg, Sweden; 3 Institute of Molecular, Cell and Systems Biology, College of Medical, Veterinary and Life Sciences, University of Glasgow, Glasgow, United Kingdom; 4 Groningen Bioinformatics Centre, Groningen Biomolecular Sciences and Biotechnology Institute, University of Groningen, Groningen, the Netherlands; 5 Biological Chemistry and Drug Discovery, College of Life Sciences, University of Dundee, Dundee, United Kingdom; 6 School of Information Systems, Computing and Mathematics, Brunel University, Uxbridge, United Kingdom; 7 Department of Molecular Cell Physiology, Faculty of Earth and Life Sciences, VU University Amsterdam, Amsterdam, the Netherlands; 8 Faculty of Life Sciences, Manchester Institute of Biotechnology, University of Manchester, Manchester, United Kingdom; 9 Department of Pediatrics and Systems Biology Centre for Energy Metabolism and Ageing, University of Groningen, University Medical Center Groningen, Groningen, the Netherlands; National Institutes of Health, United States of America

## Abstract

Dynamic models of metabolism can be useful in identifying potential drug targets, especially in unicellular organisms. A model of glycolysis in the causative agent of human African trypanosomiasis, *Trypanosoma brucei*, has already shown the utility of this approach. Here we add the pentose phosphate pathway (PPP) of *T. brucei* to the glycolytic model. The PPP is localized to both the cytosol and the glycosome and adding it to the glycolytic model without further adjustments leads to a draining of the essential bound-phosphate moiety within the glycosome. This phosphate “leak” must be resolved for the model to be a reasonable representation of parasite physiology. Two main types of theoretical solution to the problem could be identified: (i) including additional enzymatic reactions in the glycosome, or (ii) adding a mechanism to transfer bound phosphates between cytosol and glycosome. One example of the first type of solution would be the presence of a glycosomal ribokinase to regenerate ATP from ribose 5-phosphate and ADP. Experimental characterization of ribokinase in *T. brucei* showed that very low enzyme levels are sufficient for parasite survival, indicating that other mechanisms are required in controlling the phosphate leak. Examples of the second type would involve the presence of an ATP:ADP exchanger or recently described permeability pores in the glycosomal membrane, although the current absence of identified genes encoding such molecules impedes experimental testing by genetic manipulation. Confronted with this uncertainty, we present a modeling strategy that identifies robust predictions in the context of incomplete system characterization. We illustrate this strategy by exploring the mechanism underlying the essential function of one of the PPP enzymes, and validate it by confirming the model predictions experimentally.

## Author Summary

Mathematical models have been valuable tools for investigating the complex behaviors of metabolism. Due to incomplete knowledge of biological systems, these models contain inevitable uncertainty. This uncertainty is present in the measured or estimated parameter values, but also in the structure of the metabolic network. In this paper we increase the coverage of a particularly well studied model of glucose metabolism in *Trypanosoma brucei*, a tropical parasite that causes African sleeping sickness, by extending it with an additional pathway in two compartments. During this modeling process we highlighted uncertainties in parameter values and network structure and used these to formulate new hypotheses which were subsequently tested experimentally. The models were improved with the experimentally derived data, but uncertainty remained concerning the exact topology of the system. These models allowed us to investigate the effects of the loss of one enzyme, 6-phosphogluconate dehydrogenase. By taking uncertainty into account, the models demonstrated that the loss of this enzyme is lethal to the parasite by a mechanism different than that in other organisms. Our methodology shows how formally introducing uncertainty into model building provides robust model behavior that is independent of the network structure or parameter values.

## Introduction

Human African trypanosomiasis, or sleeping sickness, is a lethal disease caused by the protozoan parasite *Trypanosoma brucei* [1], [2]. Current drugs against trypanosomiasis are difficult to administer, unacceptably toxic, and relatively expensive when considering the impoverished state of most patients [3]. The search for new promising drug targets has been supported by a series of computational models, focusing on energy production via the glycolytic pathway [4]–[8]. This pathway has been considered to be a particularly promising target for new drugs, as a 50% reduction in ATP production in *T. brucei* by inhibitors of glycolysis was demonstrated to be sufficient to kill the parasites [9].

In this paper, we extended this model of glycolysis with a description of the pentose phosphate pathway (PPP), another essential [10], [11] maintenance pathway that generates reducing equivalents in the form of NADPH that are used in the cells' protection against oxidative stress and thus provides a metabolic link to another important drug target in trypanosomes [12]. Bloodstream form *T. brucei* relies on the PPP as the primary source of NADPH, in contrast to procyclic *T. brucei* which can also produce NADPH via malic enzyme [13].

Kinetic models of metabolism, such as those constructed of *T. brucei* glycolysis, include kinetic parameters that are uncertain for a variety of reasons, such as measurement errors or lack of data. We have previously shown the importance of taking uncertainty of these parameter values into account [8]. This approach can demonstrate which model behaviors are robust to the uncertainty, helping to indicate fragilities in the model and highlighting areas in need of more detailed experimental characterization. When introducing the PPP to the model of glycolysis we face the additional challenge of uncertainty in the model topology itself; we do not know for sure which reactions are active in the context of this pathway. Again an explicit consideration of this uncertainty in the model construction reveals robust behavior and indicates gaps in our knowledge of *T. brucei* metabolism. We illustrate the consequences of this uncertainty, and the robust predictions that are still possible, by exploring model behavior under various physiological conditions (different levels of oxidative stress and of external glucose concentration) and characterizing the effects of the inhibition of 6-phosphogluconate dehydrogenase (6PGDH), an essential enzyme of the pentose phosphate pathway, proposed as another potential drug target [14].

## Results

### Simple modeling of the PPP creates a glycosomal phosphate “leak”

**Figure 1:**
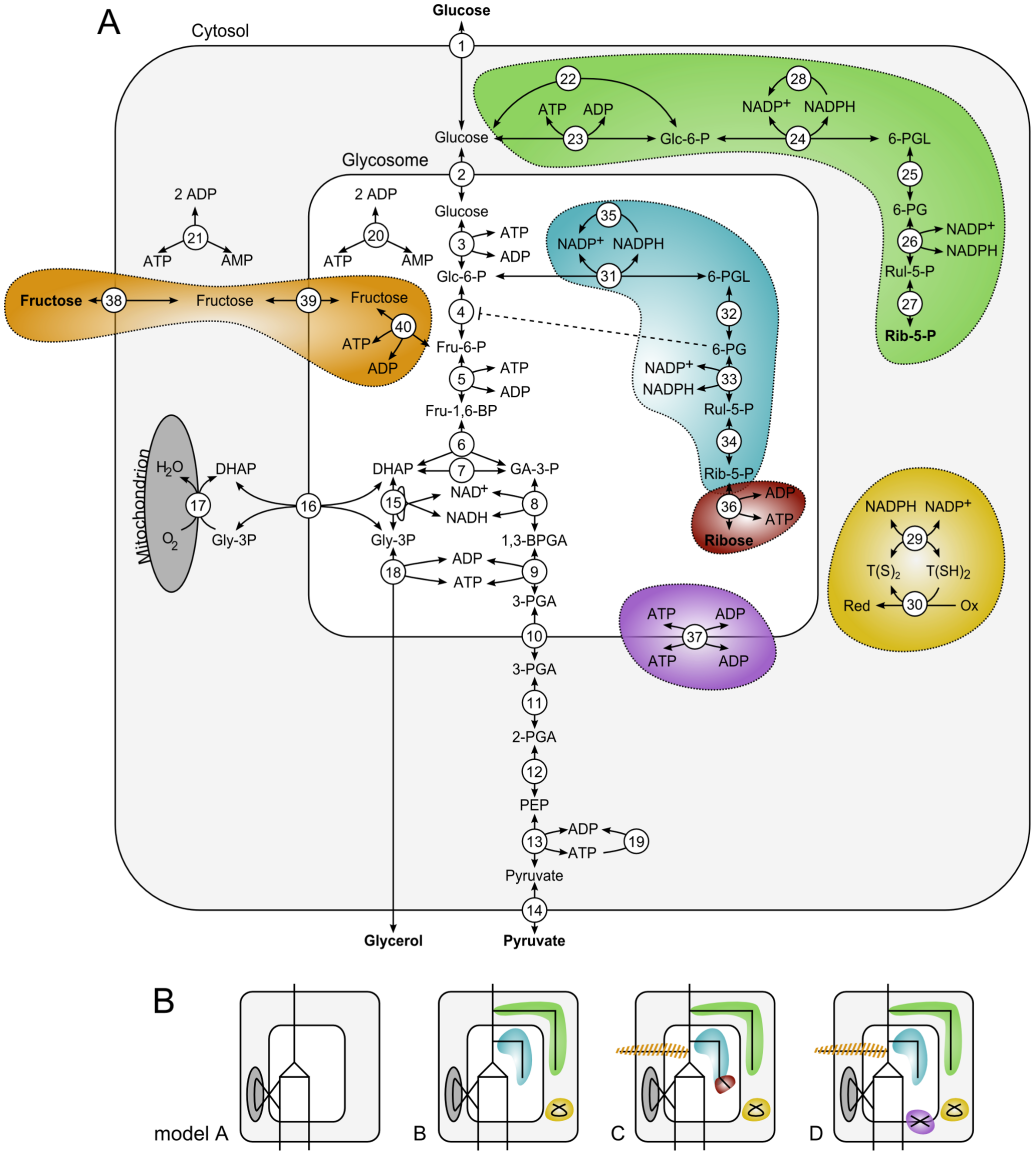
Schematic overview of the models used for simulation. (*A*) Detailed scheme of the modeled metabolic pathways. The numbered arrows correspond to reactions from Table 1. Extensions to the original model of glycolysis are indicated by colored shapes. Boundary metabolites are in bold, glycosomal Rib-5-P is a boundary metabolite in model C and D. (*B*) Schematic overview of the different models, each consisting of a unique combination of the colored modules described in (*A*) and Table 1. Model C and D can alternatively utilize fructose (model C^fru^ and D^fru^), but this branch is switched off unless specifically mentioned.

The existing mathematical model of glycolysis in *T. brucei* [8] was extended to include the reactions from the pentose phosphate pathway (PPP). In *T. brucei*, the PPP is localized to both the cytosol and glycosomes, based on subcellular localization studies [15]–[17], proteomic analysis [18], [19] and subcellular targeting-sequence-based predictions [20]. Figure 1 gives an overview of the models considered in this paper, while the stoichiometry and kinetic parameters are described in Table 1 and S1, respectively.

**Table 1:** Model stoichiometry by modules and their subsequent reactions.

Module	No.	Reaction name	Reaction stoichiometry
Glycolysis	1	GlcT_plasma membrane_	**Glc** ***_out_***↔Glc*_c_*
	2	GlcT_glycosomal membrane_	Glc*_c_*↔Glc*_g_*
	3	HXK*_g_*	Glc*_g_*+ATP*_g_*↔Glc-6-P*_g_*+ADP*_g_*
	4	PGI	Glc-6-P*_g_*↔Fru-6-P*_g_*
	5	PFK	Fru-6-P*_g_*+ATP*_g_*↔Fru-1,6-BP*_g_*+ADP*_g_*
	6	ALD	Fru-1,6-BP*_g_*↔DHAP*_g_*+GA-3-P*_g_*
	7	TPI	DHAP*_g_*↔GA-3-P*_g_*
	8	GAPDH	GA-3-P*_g_*+NAD^+^ *_g_*+**Pi** ***_g_***↔1,3-BPGA*_g_*+NADH*_g_*
	9	PGK	1,3-BPGA*_g_*+ADP*_g_*↔3-PGA*_g_*+ATP*_g_*
	10	PGAT	3-PGA*_g_*↔3-PGA*_c_*
	11	PGAM	3-PGA*_c_*↔2-PGA*_c_*
	12	ENO	2-PGA*_c_*↔PEP*_c_*
	13	PYK	PEP*_c_*+ATP*_c_*↔Pyr*_c_*+ADP*_c_*
	14	PyrT	Pyr*_c_*→**Pyr** ***_out_***
	15	GDH	DHAP*_g_*+NADH*_g_*↔NAD^+^ *_g_*+Gly-3-P*_g_*
	16	DHAP:Gly-3-P antiporter	DHAP*_c_*+Gly-3-P*_g_*↔DHAP*_g_*+Gly-3-P*_c_*
	17	GPO	Gly-3-P*_c_*+**0.5 O_2_**→DHAP*_c_*
	18	GK	Gly-3-P*_g_*+ADP*_g_*↔**Gly** ***_out_***+ATP*_g_*
	19	ATP utilization	ATP*_c_*→ADP*_c_*
	20	AK*_g_*	2 ADP*_g_*↔ATP*_g_*+AMP*_g_*
	21	AK*_c_*	2 ADP*_c_*↔ATP*_c_*+AMP*_c_*
Cytosolic extension	22	G6P utilization	Glc-6-P*_c_*↔Glc*_c_*+**Pi** ***_c_***
	23	HXK*_c_*	Glc*_c_*+ATP*_c_*↔Glc-6-P*_c_*+ADP*_c_*
	24	G6PDH	Glc-6-P*_c_*+NADP^+^ *_c_*↔6-PGL*_c_*+NADPH*_c_*
	25	PGL	6-PGL*_c_*↔6-PG*_c_*
	26	6PGDH	6-PG*_c_*+NADP^+^ *_c_*↔Rul-5-P*_c_*+NADPH*_c_*+**CO_2_**
	27	PPI	Rul-5-P*_c_*↔**Rib-5-P** ***_c_***
	28	NADPH utilization	NADPH*_c_*↔NADP^+^ *_c_*
	29	TR	TS_2,*c*_+NADPH*_c_*↔T(SH)_2,c_+NADP^+^ *_c_*
	30	TOX	T(SH)_2,c_→TS_2,*c*_
Glycosomal extension	31	G6PDH	Glc-6-P*_g_*+NADP^+^ *_g_*↔6-PGL*_g_*+NADPH*_g_*
	32	PGL	6-PGL*_g_*↔6-PG*_g_*
	33	6PGDH	6-PG*_g_*+NADP^+^ *_g_*↔Rul-5-P*_g_*+NADPH*_g_*+**CO_2_**
	34	PPI	Rul-5-P*_g_*↔**Rib-5-P** ***_g_***
	35	NADPH utilization	NADPH*_g_*↔NADP^+^ *_g_*
Model C extension	36	RK	Rib-5-P*_g_*+ADP*_g_*↔**Rib** ***_g_***+ATP*_g_*
Model D extension	37	ATP:ADP antiporter	ATP*_c_*+ADP*_g_*↔ATP*_g_*+ADP*_c_*
Fructose growth	38	FruT_plasma membrane_	Fru*_out_*↔Fru*_c_*
	39	FruT_glycosomal membrane_	Fru*_c_*↔Fru*_g_*
	40	HXK(fru)*_g_*	Fru*_g_*+ATP*_g_*↔Fru-6-P*_g_*+ADP*_g_*

Bold characters indicate external metabolites which are kept fixed. Other metabolites are internal variables of the system.

To provide glucose 6-phosphate (Glc-6-P) to the cytosolic glucose 6-phosphate dehydrogenase (G6PDH), a trace amount of cytosolic hexokinase was added in models B–D. An alternative solution, where transport of Glc-6-P over the glycosomal membrane is allowed, gave similar results. Fractionation studies that suggest an exclusively glycosomal localization of hexokinase activity [21] are insufficiently sensitive to discriminate a trace amount of cytosolic hexokinase. Moreover, enzymes targeted to the glycosome can be fully folded in the cytosol, which leaves room for a small, residual activity in this compartment. We therefore decided to explore the model behavior in the presence of cytosolic hexokinase in the following analyses, yet acknowledge that this choice is somewhat arbitrary. A reaction utilizing cytosolic Glc-6-P was added in models B–D, as absence of this reaction resulted in accumulation of Glc-6-P in the cytosol (25% of the models have a cytosolic Glc-6-P concentration over 20 mM, in contrast to a measured steady-state value of 2.6 mM). This reaction primarily represents the use of Glc-6-P in other cytosolic pathways, such as the glycosylation of proteins [22].

**Figure 2:**
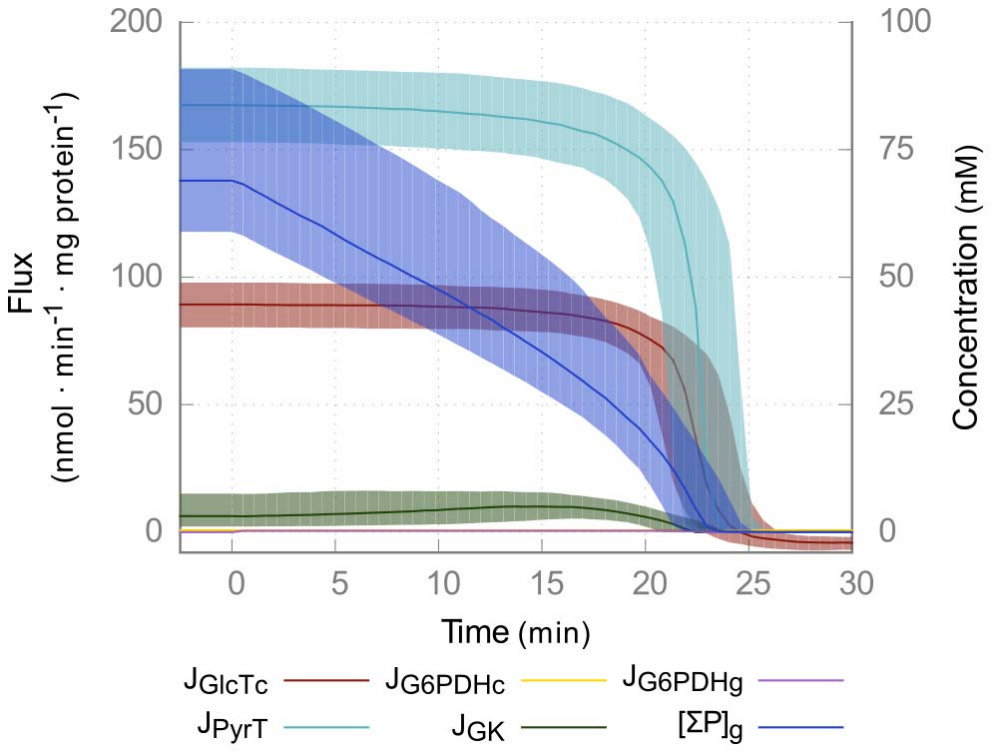
Phosphate leak in model with PPP. Time course simulation of model B, in which the reactions of the glycosomal PPP are switched on at *t* = 0 by increasing their V_max_ value from zero to the value given in Table S1. Glc_e_ is 5 mM and *k_TOX_* = 2 µl·min^−1^·mg protein^−1^. Solid lines indicate medians, shaded areas show interquartile ranges. Fluxes (J) are plotted on the left y-axis and are indicative of glucose uptake (GlcT_plasma membrane_), glycerol (GK) and pyruvate production (PyrT) and the two branches of pentose phosphate pathways (G6PDH_c/g_). The sum of bound phosphates in the glycosome (ΣP_g_), as exists in the model of glycolysis (Table 2, moiety 5), is plotted on the right y-axis. Within 25 minutes, all bound phosphates within the glycosome are depleted and all metabolic fluxes subsequently drop to zero.

The model of glycolysis (Figure 1B, model A) has a conserved sum of phosphorylated metabolites in the glycosome, consisting of ATP, ADP and glycolytic intermediates (Table 2). Extension of the glycolysis model with the PPP (Figure 1B, model B) introduces a drain from this moiety of phosphorylated glycosomal compounds. While the extension with the PPP adds metabolites in the glycosome that are derived from Glc-6-P, these phosphorylated PPP intermediates are prevented from re-entering glycolysis as two key enzymes of the non-oxidative PPP—ribulose-5-phosphate epimerase [23] and transketolase [17], [23]—are not expressed in the bloodstream form of the parasite. This renders ribose 5-phosphate (Rib-5-P) as the end product of the PPP according to the classical scheme, and leads to a lethal drainage of phosphates from the previously conserved moiety (Figure 2). This phosphate “leak” only entails bound phosphate, and not inorganic phosphate, which is independent of the conserved moiety. This leak of phosphates from the conserved moiety of bound phosphates is only observed in the glycosome, as the bound phosphates are not conserved in the cytosol, where there is a net production of ATP via pyruvate kinase. Model B is therefore an obviously non-physiological model and was not investigated further. The phosphate leak is an effect of the stoichiometry of the model and not the enzyme kinetics: the formal proof of this is that there exists no elementary mode (Table S2) for model B with a flux through the PPP.

**Table 2:** Conserved moieties in the four models of Figure 1.

Model	Moieties	Metabolites
A	1	ATP*_g_*+ADP*_g_*+AMP*_g_*
	2	ATP*_c_*+ADP*_c_*+AMP*_c_*
	3	NADH*_g_*+NAD^+^ *_g_*
	4	Gly3-P*_c_*+DHAP*_c_*
	5	Glc-6-P*_g_*+Fru-6-P*_g_*+2 Fru-1,6-BP*_g_*+DHAP*_g_*+GA-3-P*_g_*+1,3-BPGA*_g_*+Gly-3-P*_g_*+2 ATP*_g_*+ADP*_g_*
B	1,2,3,4	
	6	NADPH*_g_*+NADP^+^ *_g_*
	7	NADPH*_c_*+NADP^+^ *_c_*
	8	TS*_2,c_*+T(SH)*_2,c_*
C	1,2,3,4,6,7,8	
	9	Glc-6-P*_g_*+Fru-6-P*_g_*+2 Fru-1,6-BP*_g_*+DHAP*_g_*+GA-3-P*_g_*+1,3-BPGA*_g_*+Gly-3-P*_g_*+2 ATP*_g_*+ADP*_g_*+6-PG*_g_*+6-PGL*_g_*+Rul-5-P*_g_*+Rib-5-P*_g_*
D	1,2,3,4,6,7,8	

Where moieties are present in multiple models, only the number is indicated. Moieties 1–4 are conserved in all four model versions. Moieties 6–8 are a result of the extension with the pentose phosphate pathway. Moiety 9 is a modified version of moiety 5, now including the phosphorylated metabolites of the pentose-phosphate pathway. The latter appears when the glycosomal PPP is completed with ribokinase. When the glycosomal PPP is linked to ATP transport instead, moiety 5 disappears altogether.

### Exploring solutions to the phosphate leak

There is uncertainty as to how this glycosomal phosphate leak is prevented in the parasite. Two types of solution can be considered: (i) maintaining the conserved moiety of phosphorylated metabolites by including additional reactions in the glycosome, or (ii) transferring phosphorylated metabolites over the glycosomal membrane. No *a priori* knowledge exists on balancing of bound phosphates in the glycosome that would convincingly support either solution over the other.

### First type of solution: Maintaining the conserved moiety

For the first type of solution, a stoichiometric analysis of all known and predicted glycosomal enzymes [18]–[20] was performed to indicate what additional reactions in the glycosome can recover the bound-phosphate lost via Rib-5-P (Tables S3, S4, Figure S1). Only ribokinase, which has a C-terminal PTS-1 sequence that localizes it to the glycosome, is able to support a steady-state flux through the PPP without the requirement of additional reactions that are not known or predicted to be localized to the glycosome. The addition of hypothetical reactions in the glycosome can provide alternative solutions to the phosphate leak, however, the ribokinase solution is representative of this class of solutions where the bound phosphate is balanced within the glycosome and the moiety of bound phosphates is conserved. This solution requires ribokinase to operate in the direction of ribose and ATP production, which is not favored thermodynamically in standard conditions. With an equilibrium constant of 0.0036 (in the direction of ribose formation, [24]) ribokinase would require an almost 300-fold accumulation of Rib-5-P over ribose in the glycosome, indicating that ribose in the glycosome would be in the µM range and Rib-5-P in the mM range. This ratio can be further improved by a low ATP/ADP ratio. A similar solution, however, has already been exploited with regard to glycerol kinase at the onset of anaerobiosis. In response to loss of the mitochondrial alternative oxidase reaction under these conditions, trypanosomal glycerol kinase runs in the direction of glycerol and ATP production, as glycerol 3-phosphate accumulates and a low ATP/ADP ratio is maintained in the glycosome [25].

Model B was therefore extended with a glycosomal ribokinase, generating model C (Figure 1), which represents a solution where the glycosomal conserved moiety of bound phosphates is restored (Table 2, moiety 9). In order to provide kinetic parameters for model C, the enzyme kinetics of heterologously expressed *T. brucei* ribokinase were characterized and used to update the model (see Table S1 for determined kinetic parameters). Dynamic simulations of the extended model subsequently indicated the feasibility of ribokinase as a solution, since it allows metabolic fluxes through all branches (Figure S2 and Table S2).

When ribokinase is indeed responsible of maintaining the phosphate balance in the glycosomes, its presence is expected to be essential for parasite survival. To investigate this hypothesis, a genetic mutant (RK^RNAi^) was generated to knockdown the levels of ribokinase transcript by RNA interference. However, no growth phenotype was observed (Figure S3). Attempts to generate a gene knockout of ribokinase, surprisingly, failed in spite of numerous rounds of transfection. A conditional knockout was generated with inducible expression of an exogenous copy of the *T. brucei* ribokinase gene. While non-induced growth resulted in depletion of ribokinase, again no growth phenotype could be observed, even if the ectopic copy was switched off. It was concluded that ribokinase activity is essential but that residual levels in RK^RNAi^ and leaky expression in the conditional knockout were sufficient to fulfill this essential role.

Additionally, the requirement of a low ribose/Rib-5-P ratio to catalyze the formation of ribose by ribokinase predicts that ribose should be toxic to the parasites (Figure S4), in a similar way to glycerol that can prevent glycerol kinase from working in the reverse direction. However, incubation of parasites with ribose up to 30 mM for over 72 hours was non-lethal. From these results and the genetic mutants, it was concluded that ribokinase is unlikely the sole glycosomal enzyme responsible for maintaining the phosphate balance. Other kinases, such as arginine kinase [26] and phosphoenolpyruvate carboxykinase (PEPCK), are also present within the glycosome and bound phosphate homeostasis could involve a large and redundant network of contributing metabolic pathways. While uncertainty remains on which enzymes would be involved in preventing the phosphate leak by restoring the conserved moiety of phosphorylated metabolites, model C represents a generic representation of such a solution. Continued analysis of this model can, therefore, still provide insight into the effects of such a solution even in the absence of a specific individual enzyme.

### Second type of solution: Transferring phosphates over the glycosomal membrane

An alternative solution to the phosphate leak requires transferring bound-phosphates over the glycosomal membrane. This can be achieved by the presence of a single ATP:ADP antiporter operative across the glycosomal membrane, analogous to the yeast peroxisomal adenine nucleotide transporter [27]. Model D was generated by the addition of an ATP:ADP antiporter to model B (Figure 1), and stoichiometric analysis of model D showed the absence of a conserved moiety of phosphorylated glycosomal metabolites (Table 2).

**Figure 3:**
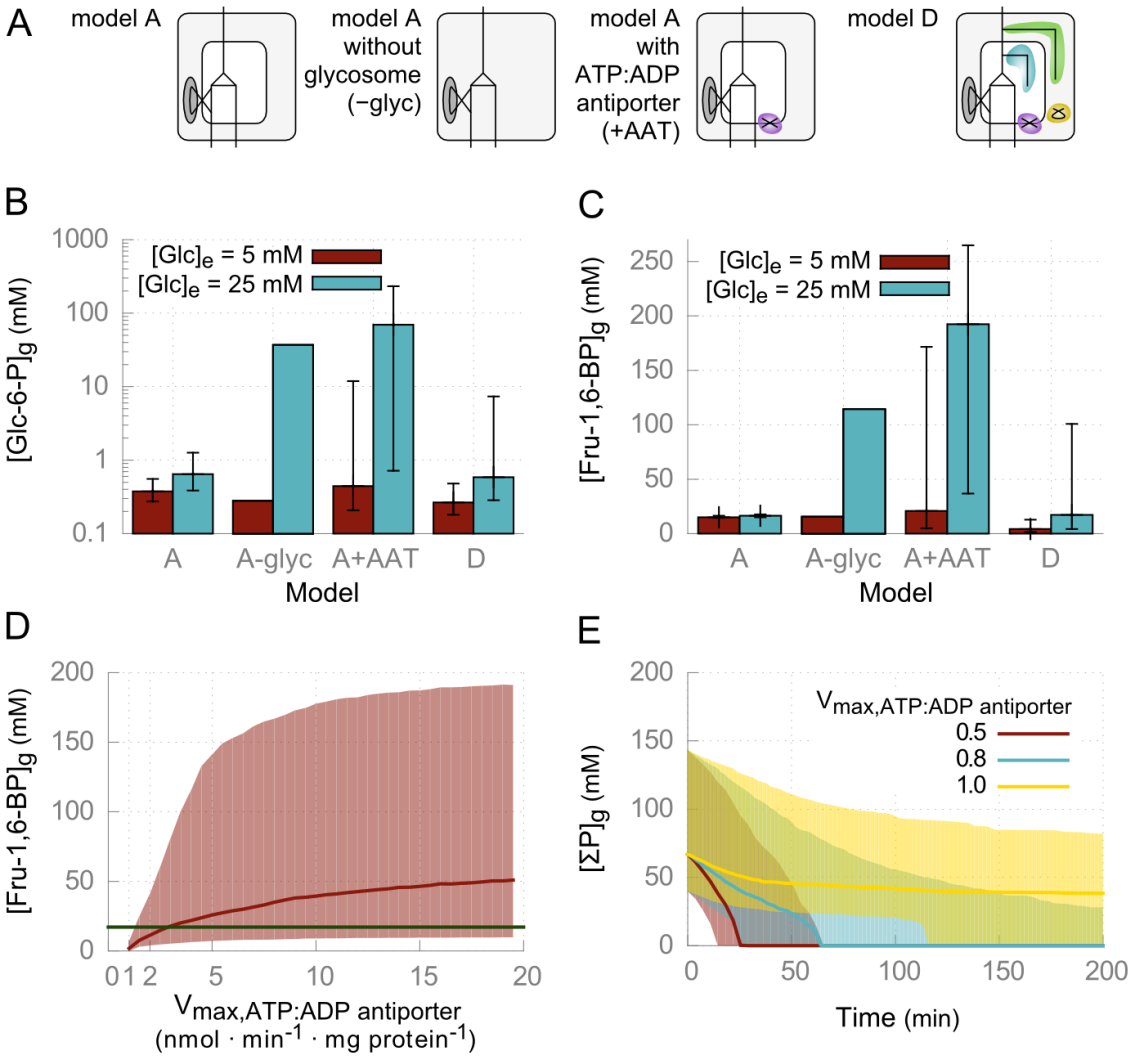
ATP:ADP antiporter mimics turbo-state. (*A*) Overview of the models used in this figure. Model A and D are from Figure 1, model A–glyc is model A without glycosomal localization, as described in [31], model A+AAT is model A with an ATP:ADP antiporter. (*B–C*) Steady-state concentrations of glycosomal Glc-6-P and Fru-1,6-BP are depicted in the various models. (*D*) Increasing the activity of the ATP:ADP antiporter (V_max,ATP:ADP antiporter_) in model D leads to a high risk of accumulation of hexose phosphates. The green line indicates the concentration of Fru-1,6-BP in the original model of glycolysis (17.2 mM, panel C, model A). Glc_e_ in this simulation is 25 mM. (*E*) Time course simulation of model D at 25 mM Glc_e_ and various values for the V_max,ATP:ADP antiporter_ parameter. Plotted is the concentration of glycosomal phosphates (ΣP similar as in Figure 2, moiety 5 in Table 2). ATP:ADP antiporter activity values below 1 nmol·min^−1^·mg protein^−1^ result in depletion of glycosomal phosphates (cf. Figure 2). *k_TOX_* = 2 µl·min^−1^·mg protein^−1^ in all models. Solid lines indicate medians, shaded areas and error bars show interquartile ranges, as derived from the uncertainty modeling.

Dynamic analysis of model D showed that the absence of the conservation constraint on phosphorylated metabolites in the glycosome introduced a risk of high accumulation of these metabolites. The autocatalytic design of glycolysis means that surplus ATP produced in the later steps of glycolysis can fuel the first reactions in a positive feedback loop. This “turbo design” [28] is controlled in most organisms by additional negative feedback regulation, e.g. inhibition of PFK by PEP [29]. Trypanosomes lack such regulation, but dynamic modeling supported by direct experimentation revealed that the glycosomal localization of part of glycolysis prevents the turbo effect in trypanosomes [30], [31]. Glucose is predicted to be lethal for glycosome deficient bloodstream forms [32], while procyclic forms, which can grow on both glucose and proline, could be rescued by the removal of sugars from the medium [32], or by the ablation of hexokinase [33]. Simulations of model D indicate that the presence of an ATP:ADP antiporter mimics the absence of a glycosome, with a high risk of sugar-phosphate accumulation (Figure 3). While the presence of the PPP can partly rescue a model with the ATP:ADP antiporter from accumulation of sugar-phosphates (Figure 3B–C, compare model A+AAT and D), a high risk of accumulation remains when the antiporter is too active (Figure 3D–E). It has been shown experimentally in procyclic trypanosomes that such accumulation occurs only when enzymes are mislocalized, and this is detrimental for the cells [31]. Other types of transporters, including recently described pores in the glycosomal membrane [34], facilitating a net transport of phosphorylated compounds into the glycosome gave similar results, while the absence of known genes encoding these transporters impedes further testing of this hypothesis by genetic manipulation.

The two hypothesized solutions to the phosphate leak are both theoretically able to restore a glycolytic and PPP flux. Although current available knowledge is insufficient to explicitly exclude one of the two types of solutions, further analysis of the two models can still give us valuable insights on for instance 6PGDH inhibition, while not ignoring the uncertainty involved. We demonstrate this below.

### Predictive behavior using the extended models of trypanosome glucose metabolism

**Figure 4:**
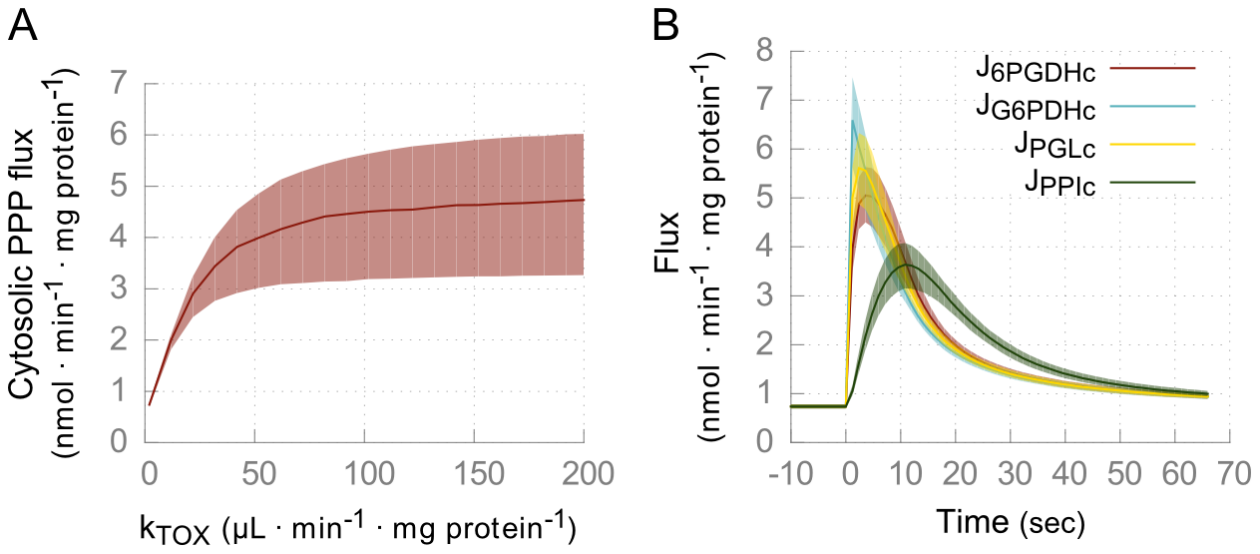
Simulations of oxidative stress in model C. (*A*) The steady state flux through the cytosolic pentose phosphate pathway in model C as a function of the oxidative stress by varying the kinetic constant *k_TOX_*. (*B*) Fluxes through the cytosolic PPP enzymes as a function of time upon sudden oxidative stress. During the whole time-course, *k_TOX_* = 2 µl·min^−1^ · mg protein^−1^. The system is removed from steady state at *t* = 0, by setting 99% of the NAD(P)H and trypanothione pools to the oxidized form. Shown is the relaxation of the cytosolic PPP fluxes. Solid lines indicate medians, shaded areas show interquartile ranges. Near identical results were obtained for model D (Figure S5).

Dynamic simulations using both models, containing either of the two theoretical solutions to the phosphate-leak problem, showed that oxidative stress regulates the flux through the PPP (Figure 4A), in line with measured increases in the flux through the PPP in the related species *T. cruzi* upon induced oxidative stress [35]. Low oxidative stress (*k_TOX_* = 2 µL•min^−1^•mg protein^−1^) represents a healthy proliferating trypanosome and results in a low PPP flux, supported by the experimental observation that almost all consumed glucose is excreted as either pyruvate or glycerol [36], [37]. A sudden burst of oxidative stress was simulated by almost completely (99%) oxidizing the pools of trypanothione and NADPH and subsequently allowing the model to reach steady state. The parasite was able to restore its redox balance in about one minute (Figure 4B). This experiment simulates an extreme level of oxidative stress, but if in reality the pools never become 99% oxidized, the PPP can respond even faster.

It has previously been proposed that inhibition of the third enzyme of the PPP, 6-phosphogluconate dehydrogenase (6PGDH), should be lethal to trypanosomes [38]. In Drosophila [39], [40], yeast [41] and mammalian cancer cells [42], lethality of 6PGDH inhibition is based on the inhibition of glycolysis by the accumulation of 6-phosphogluconate (6-PG). Inhibition of 6PGDH results in accumulation of its substrate 6-PG, and as a potent inhibitor of phosphoglucose isomerase (PGI) [43], 6-PG subsequently inhibits the conversion from glucose 6-phosphate (Glc-6-P) to fructose 6-phosphate. With Glc-6-P then instead directed towards the PPP, 6-PG accumulates to even higher levels, ultimately inhibiting the ATP producing glycolytic pathway. In Drosophila and yeast, this lethal phenotype can be rescued by additional loss of G6PDH, completely shutting down the PPP and as such preventing accumulation of 6-PG [40], [41]. Growth on alternative hexoses such as fructose, bypassing the PGI reaction and as such preventing inhibition of glycolysis, was shown to rescue 6PGDH deficient cancer cells [42]. A similar route to lethality of 6PGDH inhibition via PGI inhibition was hypothesized for *T. brucei* [10], but no experimental proof exists to corroborate this hypothesis. Alternatively, the reduced PPP flux in 6PGDH inhibited trypanosomes potentially renders the parasites more susceptible to oxidative stress. To analyze these two hypotheses, the activity of 6PGDH was investigated in both models C and D.

**Figure 5:**
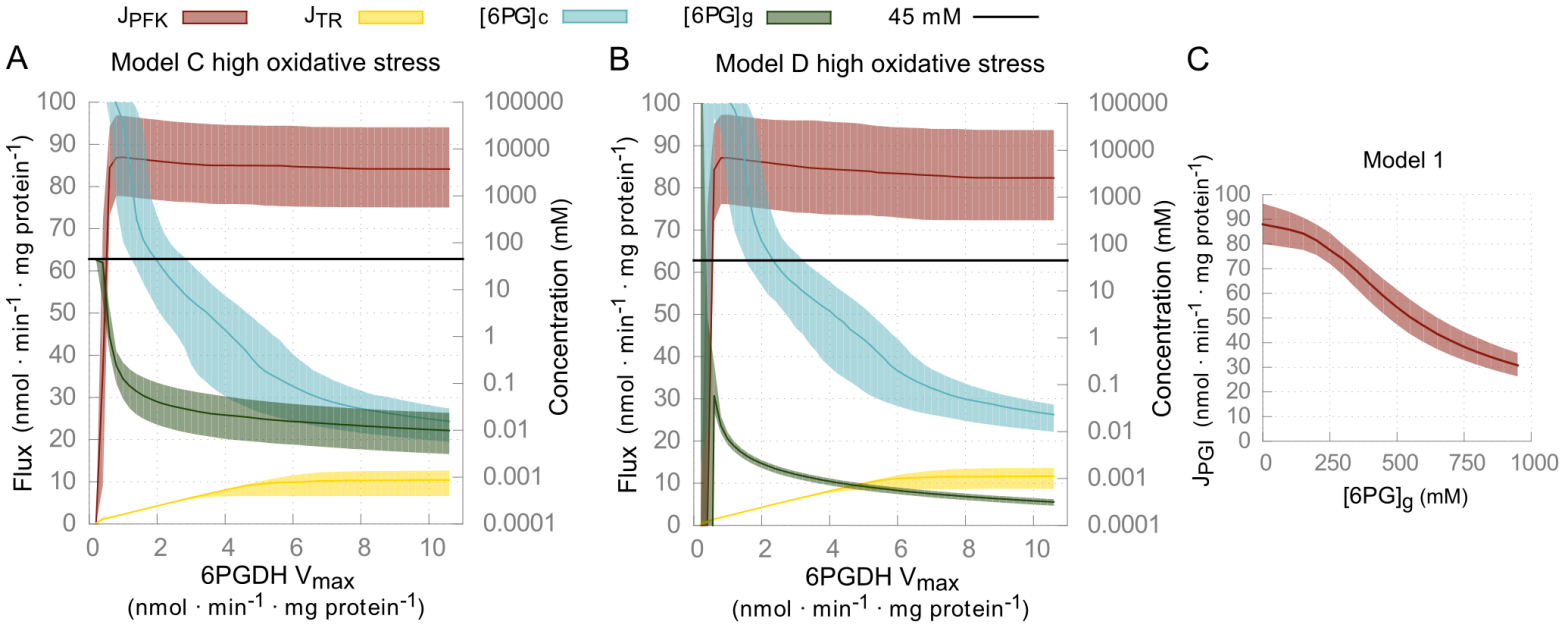
Simulations of 6PGDH inhibition and 6-PG accumulation. (*A–B*) The effects of inhibition of 6PGDH on 6-PG concentrations and metabolic fluxes were simulated by reducing *V*_max,6PGDH_ in model C and D at high oxidative stress (*k_TOX_* = 200 µl·min^−1^·mg protein^−1^). Simulations at low oxidative stress (*k_TOX_* = 2 µl·min^−1^·mg protein^−1^) are shown in Figure S6. ATP production flux as steady-state flux through PFK is indicated in red, while trypanothione reductase steady-state flux is indicated in yellow, both plotted on the left y-axis. Steady-state concentration of cytosolic (blue) and glycosomal (green) 6-phosphogluconate are plotted on the right y-axis. Shaded areas indicate interquartile ranges. (*C*) Steady-state flux through glycolysis as a function of the glycosomal 6-PG concentration in model A. A glycosomal 6-PG concentration of around 500 mM reduces the glycolytic flux by 50%.

6PGDH inhibition in both models resulted in the accumulation of 6-PG as anticipated (Figure 5A–B). Both models gave very similar results, as the only major difference is that glycosomal 6-PG in model C is restricted to 45 mM due to the constraint of the conserved moiety of phosphorylated metabolites. Highly oxidative conditions resulted in a cytosolic 6-PG accumulation that started at lower 6PGDH inhibitions, as the cytosolic PPP flux is activated to provide the NADPH required by trypanothione reductase. Only when the 6PGDH activity was decreased by more than 95% was glycolysis reduced to a negligible flux. To observe the effects of accumulated 6-PG on PGI in isolation from other effects, model A with a 6-PG sensitive PGI was simulated at different 6-PG levels. A 50% reduction in glycolytic flux is lethal to the parasite [9], but this level of inhibition was reached only at 6-PG levels of around 500 mM (Figure 5C). In model C, the conserved moiety of bound phosphates restrict the glycosomal 6-PG level to only 45 mM (Figure 5A), while in model D such high accumulation of glycosomal 6-PG occurs in combination with far more extreme accumulation of cytosolic 6-PG (Figure 5B). Neither model therefore supports the PGI inhibition hypothesis as the explanation of the lethality 6PGDH inhibition in *T. brucei*.

The second hypothesis initially proposed posits that 6PGDH inhibition is lethal due to the reduced flux through the PPP and the associated increased sensitivity for oxidative stress. In both models, the simulation of 6PGDH inhibition demonstrates how the cell's ability to cope with oxidative stress is strongly affected, as the flux through trypanothione reductase is inhibited when the activity of 6PGDH is reduced by more than 50% (Figure 5A–B). A third hypothesis introduced in model C reveals that the accumulation of glycosomal 6-PG depletes other bound phosphates within the glycosome, thus inhibiting the glycolytic flux. Sensitivity to oxidative stress, and the depletion of bound phosphates from the conserved moiety, could therefore both explain loss of viability due to ablation of 6PGDH. While accumulation of the toxic lactone precursor of 6-PG through product inhibition of the lactonase enzyme is another possibility, the high equilibrium constant of this reaction renders it insensitive to its product [44]. Finally, a reduced production of the nucleotide precursor Rib-5-P by the PPP could explain the 6PGDH inhibition lethality.

### RNA interference confirms that 6PGDH is essential, but lethality is not due to inhibition of glycolysis

**Figure 6:**
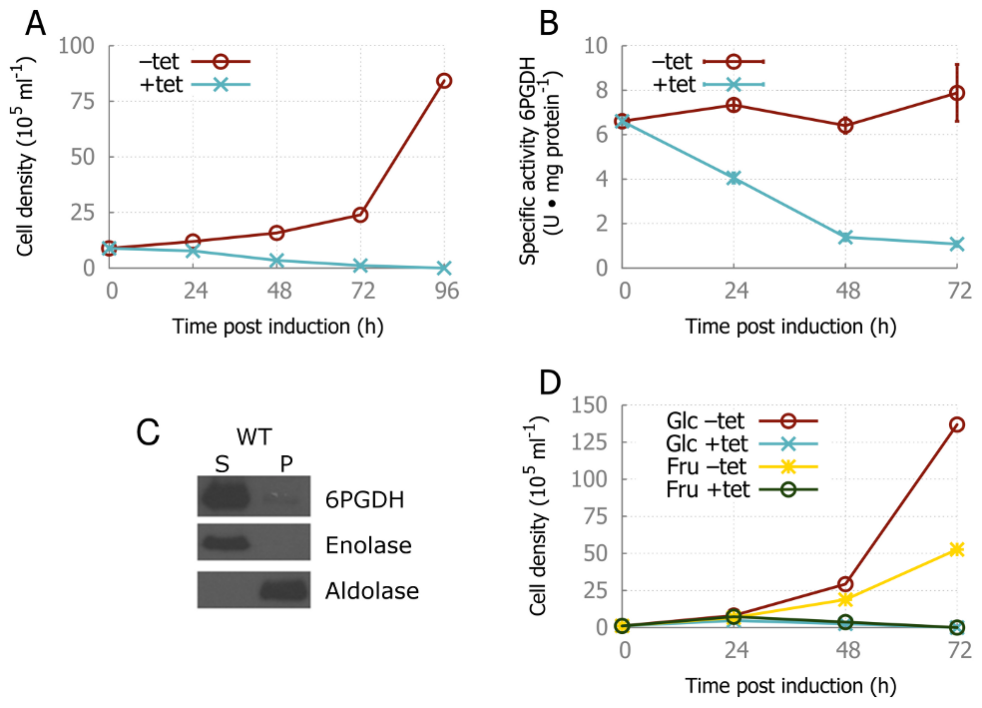
Ablation of 6PGDH. (*A*) Effect of 6PGDH ablation on the growth rate. A non-induced 6PGDH^RNAi^ culture, grown in glucose-containing HMI-9 was split at 0 h, +tet is induced with tetracycline, while −tet is the non-induced control. *(B)* Specific activities of 6PGDH in induced and control 6PGDH^RNAi^ parasites. *(C)* Western blot showing predominant co-localization of 6PGDH with the glycosomal marker aldolase (fraction S), while a faint band can also be observed in the cytosolic fraction P with the marker enolase. *(D)* Cell densities during growth on different substrates. At *t* = 0 h, a 6PGDH^RNAi^ culture grown on glucose was split at 1·10^5^ ml^−1^ to HMI-9 with either glucose or fructose, and in the absence and presence of tetracycline. Plotted cell densities are cumulative, as −tet cultures were split at 48 h to 1·10^5^ ml^−1^. A higher starting cell density was used to allow the parasites to adapt to the change in carbon source. Growth on fructose is slower than on glucose, but is unable to rescue the induced cultures.

The predictions from the modeling were tested experimentally. A genetic mutant in which the 6PGDH transcript is knocked down by RNA interference (6PGDH^RNAi^) yielded reduced levels and activities of 6PGDH (Figure 6A) and subsequent cell death, confirming the severe effects of 6PGDH inhibition observed during model simulations. Antibodies against 6PGDH also revealed this enzyme to be distributed to both cytosol and to a lesser degree the glycosome (Figure 6B).

The 6PGDH activity measurements indicate that the growth of the parasites is affected when the 6PGDH activity is reduced by less than 50% in the first 24 hours. Even after 72 hours, 15% of the 6PGDH activity remains. This suggests that the first and third hypotheses, i.e. PGI inhibition and the depletion of the bound phosphate moiety, are unlikely. 6PGDH^RNAi^ parasites could not be rescued by the addition of 0.5 mM ribose, as an alternative pathway to Rib-5-P, suggesting that the availability of Rib-5-P for nucleotide biosynthesis was not responsible for the lethal phenotype.

To test whether the proposed feedback loop related to inhibition of PGI by accumulating 6-PG we tested how the 6PGDH^RNAi^ trypanosomes behaved when grown in fructose, a glycolytic substrate [11], [37], [45] that can enter the pathway after the PGI step, which was previously shown to rescue growth of 6PGDH deficient cancer cells [42].

Model C and D were both extended using the fructose transporter and its subsequent utilizing reactions (Figure 1), using parameter values from the literature (Table S1). The rate equation of hexokinase was adapted to describe the substrate competition of glucose and fructose. Steady-state calculation of the fructose versions of the models (model C^fru^ and D^fru^) predict that growth of parasites is supported on fructose, although the lethal effect of 6PGDH inhibition could not be averted by using this substrate (Figure S7). This again indicates that the lethal effect of 6PGDH loss in trypanosomes is not due to a catastrophic positive feedback loop induced through PGI inhibition.

In vitro, 6PGDH^RNAi^ had a slightly slower growth when glucose in the medium was substituted with fructose (Figure 6C). However, in common with model predictions, growth on fructose was unable to rescue the lethal phenotype of 6PGDH ablation in the 6PGDH^RNAi^ cell line (Figure 6C). The fact that G6PDH is also essential for bloodstream form *T. brucei* [11], whereas loss of this enzyme can revert lethality associated with loss of 6PGDH in Drosophila [40] and yeast [41], further indicates that it is loss of the oxidative branch of the PPP *per se* in *T. brucei* that is responsible for death, rather than the glycolysis inhibitory loop proposed in other eukaryotes.

## Discussion

The mathematical model of glucose metabolism in the bloodstream form trypanosome is among the most extensively curated dynamic models of cellular metabolism. The model accurately simulates a range of features of glucose metabolism in the parasitic protist. In this paper, we extended this well curated model of trypanosomal glycolysis with the PPP. However, in doing so it soon becomes obvious that our knowledge of even this central part of metabolism is incomplete. Using our previously established strategy for explicitly handling uncertainty in dynamic models, and additionally taking uncertainty on the metabolic network topology into account, we are able to characterize important gaps in our knowledge of trypanosomal metabolism, but we could also show which behaviors are robust to the uncertainty.

The first type of solution to the observed phosphate “leak”, introduced upon extension of the glycolytic model with the PPP, required the presence of additional enzymatic reactions within the glycosome to recover bound phosphate. As an example of this type of solution, glycosomal ribokinase added to the kinetic model was able to restore the glycosomal phosphate balance by generating ATP. Kinetic characterization of recombinant *T. brucei* ribokinase showed that the enzyme could indeed work in the ribose production direction (albeit far less effectively than in the opposite direction). Various genetic mutants of ribokinase, however, subsequently indicated that it is unlikely that only ribokinase is involved in restoring the phosphate balance. The gene appears to be essential, but the ribokinase enzyme is apparently present in vast excess since no phenotype could be observed in cells in which the transcript had been knocked down by RNA interference. Also, in cells where an inducible copy of the gene is present in place of both endogenous alleles, growth occurs even when the gene is switched “off”, presumably due to leaky expression. The essential role for small amounts of ribokinase is unknown, but it seems unlikely to be the sole enzyme capable of sustaining the bound-phosphate balance in these cells. Given that the glycosome contains multiple pathways beyond glycolysis and the PPP, such as purine salvage, pyrimidine biosynthesis, and parts of the ether lipid, fatty acid and isoprenoid metabolic pathways (Table S3), a highly redundant system could provide a robust network to maintain phosphate balance.

A second type of solution to the bound-phosphate leak would involve a flow of bound phosphates into and out of the glycosome. As an example of this type of solution, addition of a theoretical glycosomal ADP:ATP antiporter analogous to the adenine nucleotide antiporter found in yeast peroxisomes [27] allowed glycolysis and the oxidative branch of the PPP to operate in concert, as shown by the modeling results. However, since no genes encoding the necessary transporters have been identified, experimental support of these model predictions is not possible at this point. Another variant of this type of solution would involve non-specific pores in the glycosomal membrane, working in tandem with low levels of residual glycolytic enzyme activity in the cytosol [46], but again without knowing which genes encode the glycosomal membrane translocases we remain uncertain as to any roles they might play.

Since the topology of the active metabolic network in the glycosome remains uncertain, neither of the two types of solutions models was discarded, and both models were used together to probe aspects of the behavior of the glycolytic and pentose phosphate pathways, expanding on the modeling strategy introduced in [8]. Simulations of the two model types provided very similar results; behavior under oxidative stress was nearly identical, while the inhibition of 6PGDH only showed minor differences between the two models, always taking into account the additional uncertainty about the exact kinetic parameters of all enzymes involved.

While the two types of model showed different behavior at very low 6PGDH activities, both were able to exclude the initial hypothesis that inhibition of PGI by accumulated 6-PG is the explanation for the lethal effect of 6PGDH inhibition [10], [38]. This was verified experimentally when RNA interference confirmed that loss of 6PGDH is lethal to cells and that this lethality is retained when fructose is used as the carbon and energy source. Since fructose enters the pathway after PGI, the feedback loop via PGI, proposed to operate in other eukaryotes [39]–[42], cannot explain lethality. A remaining possibility is that inhibition of 6PGDH renders the parasite more sensitive to oxidative stress, but we could not test this given that knockdown of the gene is lethal.

In this article we demonstrate how building models of metabolism can provide a powerful means to indicate where our knowledge of the biological system is lacking. We can then hypothesize possible solutions to these the gaps in our knowledge and test these possible solutions, both *in silico* and experimentally. Interestingly, even where uncertainties about the actual structure of the system cannot be resolved for technical reasons and experimental testing of the hypotheses is limited, explicit consideration of the uncertainties during model construction and analysis allows us to make meaningful predictions in the face of limited information. This considerably extends the usefulness of many dynamic models, as uncertainty can be levered as strength.

## Materials and Methods

### Stoichiometry

The computational model was constructed in a modular way. The reactions included in each module are listed in Table 1 and graphically represented in Figure 1. The starting point is a module describing glycolysis in bloodstream form *T. brucei* [7], [8]. A few modifications in the kinetic equations are listed below. There are two PPP modules, each consisting of an almost identical set of reactions; one is localized entirely in the glycosomal matrix and the other in the cytosol. To allow flux through the glycosomal PPP the model has been further extended with either a ribokinase module or an ATP:ADP antiporter across the glycosomal membrane. The rationale behind these extensions is discussed in the Results section.

Ribose 5-phosphate and ribose are modeled as end-products of the PPP, depending on model version and compartment. As no published intracellular concentrations are available for ribose 5-phosphate and ribose, their cytosolic and glycosomal concentrations were fixed at 0.01 mM unless stated otherwise.

### Kinetic equations

#### Generic equations

Most of the rate equations follow the generic form:
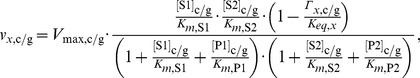
(1)for reactions with two substrates and two products. Reactions with only one substrate and product involved follow:
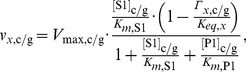
(2)where *x* identifies the reaction, S1 and S2 are the substrates while P1 and P2 are the products of the reaction. Subscripts *c/g* indicates that separate equations are used for cytosolic and glycosomal hexokinase. *K_eq_* is the standard equilibrium constant, and Γ_x,*c/g*_ specifies the ratio of substrates and products and is defined as
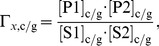
(3)in Equation 1, and
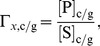
(4)in Equation 2.

#### Modified glycolysis model

The glycolysis module has been modified as compared to the latest version [7], [8] by using reversible rate equations for hexokinase (HXK), phosphofructokinase (PFK) and pyruvate kinase (PYK), which were modeled previously as irreversible reactions. Hexokinase now follows Equation 1 and 3.

PFK is modeled with reversible ordered bi-bi kinetics with mixed inhibition by fructose 1,6-bisphosphate [47]:
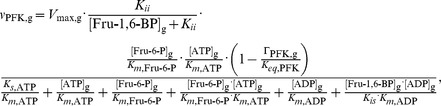
(5)where Γ_PFK,*g*_ follows Equation 3, *K_ii_* and *K_is_* describe the mixed inhibition by fructose 1,6-bisphosphate and are also taken from [47].

Pyruvate kinase is modeled reversibly with cooperativity for PEP:
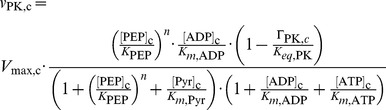
(6)where Γ_PK,*c*_ has again the same meaning as in Equation 3, and
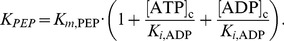
(7)


#### Phosphoglucose isomerase

The known inhibition of phosphoglucose isomerase (PGI) by the PPP intermediate 6-phosphogluconate (6-PG) was included as described in [43]:
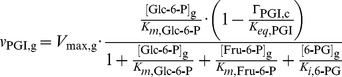
(8)where Γ_PGI,*g*_ follows equation 4.

#### ATP:ADP antiporter, G6PDH, 6PGDH, PPI, RK, TR

Most of the reversible reactions of the pentose phosphate pathway were modeled according to Equations 1–4, dependent on the number of substrates and products. For the ATP:ADP antiporter the substrates S1 and S2 are ATP_c_ and ADP_g_ and the products P1 and P2 are ATP_g_ and ADP_c_.

#### Phosphogluconolactonase

For the 6-phosphogluconolactonase (PGL) reaction, the spontaneous hydrolysis as well as the enzyme-catalyzed rate were included [48]:
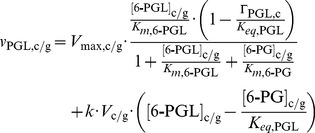
(9)where *k* is the rate of spontaneous hydrolysis and *V_c/g_* is the volume of the relevant compartment, which are 5.4549 and 0.2451 µL per mg protein for the cytosol and glycosome, respectively.

#### Glc-6-P utilization

The Glc-6-P utilization reaction was modeled as loss of Glc-6-P to glucose through a hypothetical phosphatase reaction, using Equations 2 and 4. Other fates of Glc-6-P, such as entry into inositol metabolism and mannose glycoconjugate biosynthesis, for which kinetic parameters are not known, are also covered by this generic Glc-6-P consuming reaction.

#### NADPH utilization and trypanothione oxidation reactions (TOX)

Glycosomal and cytosolic oxidation of NADPH and trypanothione are modeled with mass action kinetics:
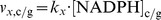
(10)where *k_x_* is the mass-action constant of reaction *x*.

### Kinetic parameters

The kinetic parameters in the rate equations mentioned above were predominantly derived from literature. If no parameters of the *T. brucei* enzyme were available, parameters from related species were used. If necessary, *K_eq_* values were corrected to pH 7. Kinetic parameters used in the fixed parameter model are listed in Table S1. Distributions of parameters used in uncertainty modeling are given in Text S1.

### Metabolite concentrations

The extension of the model with the glycosomal pentose phosphate pathway led to an extension of the conserved moiety of phosphorylated glycosomal metabolites as compared to earlier model versions [4]. For a precise overview of conserved moieties in each model version see Table 2. The new conserved sum was chosen such that extension of the glycolysis model with the glycosomal PPP and ribokinase results in similar steady state concentrations of glycolytic metabolites in both models. The conserved sum of NADPH in the cytosol and glycosome was arbitrarily chosen as 2 mM, while the conserved sum of trypanothione was chosen as 0.38 mM [12]. In the models including fructose transport, cytosolic Fru-6-P was fixed to the measured steady-state value of 0.9 mM, as additional reactions in the cytosol that are not included in the model would consume Fru-6-P. Inorganic phosphate (Pi) is not explicitly included in the model, as it is assumed to be present at saturating levels and regulated independently of glycolysis.

### Calculations and simulations

The model was initially constructed and simulations were performed in PySCeS 0.7.8 [49] or COPASI 4.8 [50]. SBML versions of the models are supplied as Dataset S1. The models in this paper have additionally been modeled while taking uncertainty into account as described in [8]. Each simulation exists of 1000 individual runs, where in each run all parameter values are sampled simultaneously. The 6PGDH *V_max_* scans of Figures 5 and S5, S6, S7, S8 show 250 simulations, as the longer time-limit and accumulating metabolites greatly increased the time for the simulation to finish (a week on a desktop PC for model D). The parameter distributions are described in more detail in Text S1, while the parameter sets are provided as Dataset S2, S3.

### Expression recombinant ribokinase

The *T. brucei* ribokinase gene (GenBank ID:3664382) was amplified from Lister 427 strain *T. brucei* genomic DNA and cloned into plasmid pET28a(+) (Novagen) to add an N-terminal His-tag. The protein was overexpressed in *E. coli* BL21(DE3), purified by Ni^2+^ chelate chromatography and dialyzed in 50 mM Tris-HCl pH 7.0, 100 mM NaCl, 20% glycerol. Purity was assessed by SDS-PAGE and Coomassie blue staining.

### Enzyme activity assays

Activity of ribokinase was assayed at 25°C in buffer containing 10 mM KH_2_PO_4_, 15 mM NaCl, 85 mM KCl and 10 mM MgCl_2_ at pH 7. Ribokinase activity in the direction of ribose 5-phosphate production was assayed spectrophotometrically according to [51], with 5 mM ribose, 5 mM phosphoenolpyruvate, 5 mM ATP, 0.2 mM NADH, 5 u PK and 5 u LDH. Ribokinase activity in the direction of ribose production was assayed spectrophotometrically with 5 mM ribose 5-phosphate, 5 mM glucose, 5 mM ADP, 0.2 mM NADP, 5 u HXK and 5 u G6PDH.

### Parasites and genetic mutants

Bloodstream form Lister 427 strain *T. brucei* parasites were routinely cultured in HMI-9 [52] at 37°C and 5% CO_2_. For growth of 6PGDH^RNAi^ on an alternative hexose source, glucose in HMI-9 was substituted with fructose at the same concentration. Antibiotic concentrations for genetic mutants were: 0.5 µg mL^−1^ phleomycin and 2.5 µg mL^−1^ hygromycin for RK^RNAi^; 0.5 µg mL^−1^ phleomycin, 5 µg mL^−1^ G418 and 10 µg mL^−1^ hygromycin for 6PGDH^RNAi^; 0.5 µg mL^−1^ puromycin for ΔRK::PAC/RK; 2.5 µg mL^−1^ hygromycin for ΔRK::HYG/RK and 0.5 µg mL^−1^ puromycin, 2.5 µg mL^−1^ hygromycin, 0.5 µg mL^−1^ phleomycin and 2 µg mL^−1^ blasticidin for ΔRK::HYG/PAC::RK^tet^. Sensitivity of *T. brucei* to ribose was assayed with alamarBlue as described previously [53].

The ribokinase knockdown cell line RK^RNAi^ was generated using the pRPa^iSL^ vector/2T1 cell line system [54], while the 6-phosphogluconate dehydrogenase (Tb427tmp.211.3180) knockdown cell line 6PGDH^RNAi^ was generated using the p2T7 vector/1313 cell line system [55]. The generation of the ribokinase knockout cell line ΔRK::HYG/PAC was attempted using constructs containing 600 bp 5′ and 3′ UTRs adjacent to the ribokinase gene, cloned into vector pTBT [56], flanking either a hygromycin phosphotransferase or puromycin *N*-acetyl-transferase gene as antibiotic resistance markers. The tetracycline-inducible expression of ribokinase in the conditional knockout ΔRK::HYG/PAC::RK^tet^ was generated by cloning the ribokinase coding sequence in pHD1336 and using cell line 449 (kind gifts by Prof Christine Clayton, Heidelberg). Parasites were transfected by Amaxa nucleofection (Lonza) [57] using Tb-BSF buffer [58], and plated out by limiting serial dilution.

### Western blotting

Polyclonal antibodies against *T. brucei* ribokinase were raised in rabbits by injection of purified recombinant *T. brucei* ribokinase, purified by protein A-chromatography, and an antibody titer of >1∶192,000 was measured by ELISA (GenicBio, Shanghai, China). The anti-serum was further purified by immunoaffinity chromatography. Recombinant *T. brucei* ribokinase was coupled to a mixture of Affi-Gel 10 and 15 (Bio-Rad). Antibodies were eluted from the column with 3.5 M MgCl_2_. Polyclonal antibodies against the *T. brucei* 6PGDH were raised in rats by infection of purified recombinant *T. brucei* 6PGDH.

For protein samples, 5·10^6^ cells were harvested by centrifugation and washed with PBS. Samples were separated on NuPAGE 4–12% Bis-Tris precast gels (Life Technologies) for 45 min at 200 V and transferred to a nitrocellulose membrane (Hybond-ECL, Amersham) at 100 V for 2 h. Membranes were blocked overnight at 4°C with 5% milk in PBS-T (PBS with 0.05% Tween-20), washed three times with PBS-T, and probed with the purified polyclonal rabbit anti-*T. brucei* ribokinase antibody, diluted 1∶2000 in 1% milk in PBS-T, for 4 h at room temperature. After three additional washes with PBS-T, the membrane was incubated with the secondary antibody (goat anti-rabbit IgG peroxidase conjugate, Calbiochem) 1∶2000 in 1% milk in PBS-T for 2 h at room temperature. After five washes with PBS-T, horseradish peroxidase activity was measured with SuperSignal HRP substrate (Novagen).

Subcellular localization of 6PGDH in trypanosomes was performed by modification of the digitonin fractionation method described in [59]. Specifically, 5·10^7^ procyclic cells were washed with STEN buffer (250 mM sucrose; 25 mM Tris-HCl, pH 7.5; 1 mM EDTA and 150 mM NaCl) and resuspended in 300 µL STEN supplemented with protease inhibitors (Roche). The cells were differentially permeabilized with 40 µg digitonin per 100 µg cellular protein (2.5·10^7^ cells) for 2 hours on ice and centrifuged at 16,000 g for two minutes. After TCA precipitation, equal amounts (equivalent of 4·10^6^ cells) of the supernatant (S) and pellet (P) were analyzed by SDS-PAGE and blotted with rat anti-Tb6PGDH. A goat anti-rabbit IgG peroxidase was used for visualization as described for ribokinase. Enolase and aldolase were used as cytosolic and glycosomal markers, respectively, and antibodies against these proteins were kindly provided by Prof. Paul Michels (University of Louvain, Belgium).

## Supporting Information

Dataset S1 **SBML versions of models.** SBML versions of the models presented in Figure 1.
(ZIP)

Dataset S2 **Parameter sets used for simulations of models A–D.** Sets of parameter values as sampled from the distributions described in Text S1, as used for simulations of models A–D during growth on glucose.
(CSV)

Dataset S3 **Parameter sets used for simulations of models C^fru^ and D^fru^.** Sets of parameter values as sampled from the distributions described in Text S1, as used for simulations of models C^fru^ and D^fru^.
(CSV)

Figure S1 **Enzymes of purine salvage pathway localized in glycosome.** The glycosomal PPP is shown with those enzymes from the purine salvage pathway that have a predicted glycosomal localization (Table S3). Reactions from the PPP model are indicated by their number from Table 1, while the additional reactions are indicated as: PRPP: phosphoribosyl pyrophosphate synthetase; AdK: adenosine kinase; ANase: adenosine nucleosidase; APRT: adenine phosphoribosyltransferase. Metabolites that are not balanced within this pathway are indicated in bold. The scheme demonstrates how the presence of the purine salvage pathway is unable to rescue the phosphate leak, as the resulting overall reaction is glucose+ATP→ribose+AMP+PPi (+CO_2_, implied to be balanced with gaseous CO_2_). Additional ADK and ANase reactions does not improve this situation, with a resulting overall reaction of glucose+ADP→2 ribose+adenine+2 PPi (+CO_2_). In contrast, ribokinase is capable of resolving the phosphate leak with a resulting overall reaction of glucose→ribose (+CO_2_).
(TIF)

Figure S2 **Steady-state fluxes through various models.** Steady state fluxes of at standard conditions (green, *k_TOX_* = 2 µl·min^−1^·mg protein^−1^) and if cytosolic PPP is maximized (red, *k_TOX_* = 200 µl·min^−1^·mg protein^−1^). Error bars indicate interquartile ranges. NA denotes ‘Not Applicable’ for branches that are absent from certain model version, Glc_e_ is 5 mM in all models. The glucose consumption flux is distributed over the production of glycerol and pyruvate and the two branches of the PPP. Note that ALD generates two trioses from every hexose, such that the fluxes through the trioses glycerol and pyruvate are double the hexose flux. The errors bars indicated interquartile ranges as a result of the uncertainty modelling. The large error bars for glycerol production are a result of its low flux and the smaller uncertainties assigned to the fluxes through the other pathways. No information is given for model B, as this model is incomplete and cannot reach a steady state (outlined in the main text).
(TIF)

Figure S3 **Genetic investigations in** ***T. brucei*** **ribokinase.** (*A*) Northern blot of RK^RNAi^ induced and non-induced *T. brucei*, samples taken 24, 48 and 72 hours post induction by tetracycline. Tubulin was used as loading control. (*B*) Western blot of RK^RNAi^ and Δrk::HYG/PAC::RK^tet^, samples taken at *t* = 0, 1, 2 and 3 days post induction by tetracycline. (*C*) Growth curve of ribokinase knockdown mutant by RNA interference. Induction of RNAi was started at t = 0 by the addition of 1 µg/mL tetracycline. Cell densities of induced (dotted line) and control (solid line) cultures were determined by cell counts, and cultures were diluted down to 2·10^4^ cells ml^−1^ at *t* = 48 and 96 h. No difference in growth effect could be observed. (*D*) Successful transfection of T. brucei with knockout constructs was confirmed by PCR.
(TIF)

Figure S4 **Ribose sensitivity.** Steady state concentrations (*A*) and fluxes (*B*) of model C at various concentrations of ribose. Solid lines indicate medians, shaded areas show interquartile ranges. Increasing ribose concentration results in a depletion of ATP and accumulation of ribose 5-phosphate in the glycosome. While the glycosomal PPP flux (J_G6PDHg_) remains mostly unaffected, the glycolytic flux (J_PGI_) is strongly reduced.
(TIF)

Figure S5 **Simulations of oxidative stress in model D.** Near identical to Figure 4, with results of model D instead. (*A*) The steady state flux through the cytosolic pentose phosphate pathway in model D as a function of the oxidative stress by varying the kinetic constant *k_TOX_*. (*B*) Fluxes through the cytosolic PPP enzymes as a function of time upon sudden oxidative stress. During the whole time-course, *k_TOX_* = 2 µl·min^−1^·mg protein^−1^. The system is removed from steady state at *t* = 0, by setting 99% of the NAD(P)H and trypanothione pools to the oxidized form. Shown is the relaxation of the cytosolic PPP fluxes. Solid lines indicate medians, shaded areas show interquartile ranges.
(TIF)

Figure S6 **Simulations of 6PGDH inhibition and 6-PG accumulation.** (*A–B*) The effects of inhibition of 6PGDH on 6-PG concentrations and metabolic fluxes were simulated by reducing *V*_max,6PGDH_ in model C and D at low oxidative stress (*k_TOX_* = 2 µl·min^−1^·mg protein^−1^). Results for simulations at low oxidative stress (*k_TOX_* = 200 µl·min^−1^·mg protein^−1^) are shown in Figure 5. ATP production flux as steady-state flux through PFK is indicated in red, while trypanothione reductase steady-state flux is indicated in yellow, both plotted on the left y-axis. Steady-state concentration of cytosolic (blue) and glycosomal (green) 6-phosphogluconate are plotted on the right y-axis. Shaded areas indicate interquartile ranges.
(TIF)

Figure S7 **Simulations of 6PGDH inhibition during growth on fructose.** (*A–B*) The effects of inhibition of 6PGDH on 6-PG concentrations and metabolic fluxes were simulated by reducing *V*_max,6PGDH_ in model C and D at high oxidative stress (*k_TOX_* = 200 µl·min^−1^·mg protein^−1^), similar to Figure 5. ATP production flux as steady-state flux through PFK is indicated in red, while trypanothione reductase steady-state flux is indicated in yellow, both plotted on the left y-axis. Steady-state concentration of cytosolic (blue) and glycosomal (green) 6-phosphogluconate are plotted on the right y-axis. Shaded areas indicate interquartile ranges.
(TIF)

Figure S8 Percentage of models reaching steady-state within 10 million simulation minutes during 6PGDH inhibitions. As explained in detail in Text S1, 250 random parameter sets were used for calculating steady-states during 6PGDH inhibition (Figures 5 and S5). Outcome of model C are shown at high (*A*) and low (*B*) oxidative stress; and in model D at high (*C*) and low (*D*) oxidative stress. Panel *A* and *C* correspond to Figures 5A–B, while panel *B* and *D* correspond to Figures S6A–B.
(TIF)

Figure S9 Percentage of models reaching steady-state within 10 million simulation minutes during 6PGDH inhibitions with growth on fructose. As explained in detail in Text S1, 250 random parameter sets were used for calculating steady-states during 6PGDH inhibition (Figures 6 and S6). Outcome at high oxidative stress are shown for model C (*A*) and D (*B*). Results from the simulations are shown in Figure S7.
(TIF)

Table S1 Kinetic parameters of the enzymes related to the PPP. Activities, which depend on the expression level of the enzymes, are specified separately for the fraction of the enzyme that is localized in the glycosome and, if applicable, for the fraction which is located in the cytosol. *c/g* indicates that cytosolic and glycosomal activities are identical. Other parameters are assumed to be identical for glycosomal and cytosolic enzyme fractions. Parameter values given here are used in the fixed parameter models. Distributions of parameter values used in uncertainty modelling are given in Text S1.
(DOCX)

Table S2 **Elementary flux modes in models of PPP.** The elementary modes of different model versions including the glycosomal PPP are listed as the overall reactions plus in brackets the individual enzyme-catalyzed reactions with their relative flux weight. For simplicity the cytosolic PPP and the cytosolic NADPH utilization were left out of this analysis. The modules included in each model version refer to Table 1 and the color-coded extensions in Figure 1 in the main text. A negative number indicates that the reaction occurs in the reverse direction as compared to Table 1 in the main text. The order of reactions corresponds to that in Table 1 in the main text. The glycolytic modes 1–3 are possible in all model versions, but are not listed again for the model versions extended with the glycosomal PPP. Elementary mode analysis of model B only results in the flux modes 1–3.
(DOCX)

Table S3 **Predicted glycosomal proteome.** All reactions present in the bloodstream form *T. brucei* glycosome, according to comprehensible glycosomal proteomics [18], [19]. Only reactions present in the bloodstream form of the parasite are included. Abbreviations and EC numbers are given for each reaction. Models are indicated where reactions are part of a model in the main text. Indicated are what reactions are part of an elementary model (see Table S4).
(DOCX)

Table S4 **Elementary modes in glycosomal proteome.** A model of glycosomal metabolism was constructed for use in METATOOL version 4.9.3 [60]. The reactions from Table S3 were used. All reactions were set as reversible, except for alternative oxidase (TAO), phosphofructokinase (PFK), fructose bisphosphatase (FBPase), and phosphogluconolactonase (PGL). Protons, Pi, PPi, H_2_O, CO_2_ and O_2_ were not included in the reactions. Glucose, 3-phosphoglyceric acid, ribose and glycerol were set as external metabolites. The resulting model has 9 elementary modes, where the first four modes are also occurring in the models described in this paper. Elementary mode 5 is a futile cycle without external metabolites involved. Elementary modes 6–9 are unlikely to occur in dividing bloodstream trypanosomes, as a high glucose to pyruvate and glycerol flux is maintained. Additionally, the activity of fructose bisphosphatase could not be measured [23], [61].
(DOCX)

Text S1 **Uncertainty modeling and distribution of parameter values.** Detailed description of the methodology for uncertainty modeling and distributions defined for sampling of random parameter values.
(DOCX)
