# Supplementary figures and images for: Handling Uncertainty in Dynamic Models: The Pentose Phosphate Pathway in Trypanosoma brucei
Source: PLoS Comput Biol. 2013 Dec 5;9(12):e1003371. doi: 10.1371/journal.pcbi.1003371 (PMC3854711; doi:10.1371/journal.pcbi.1003371)

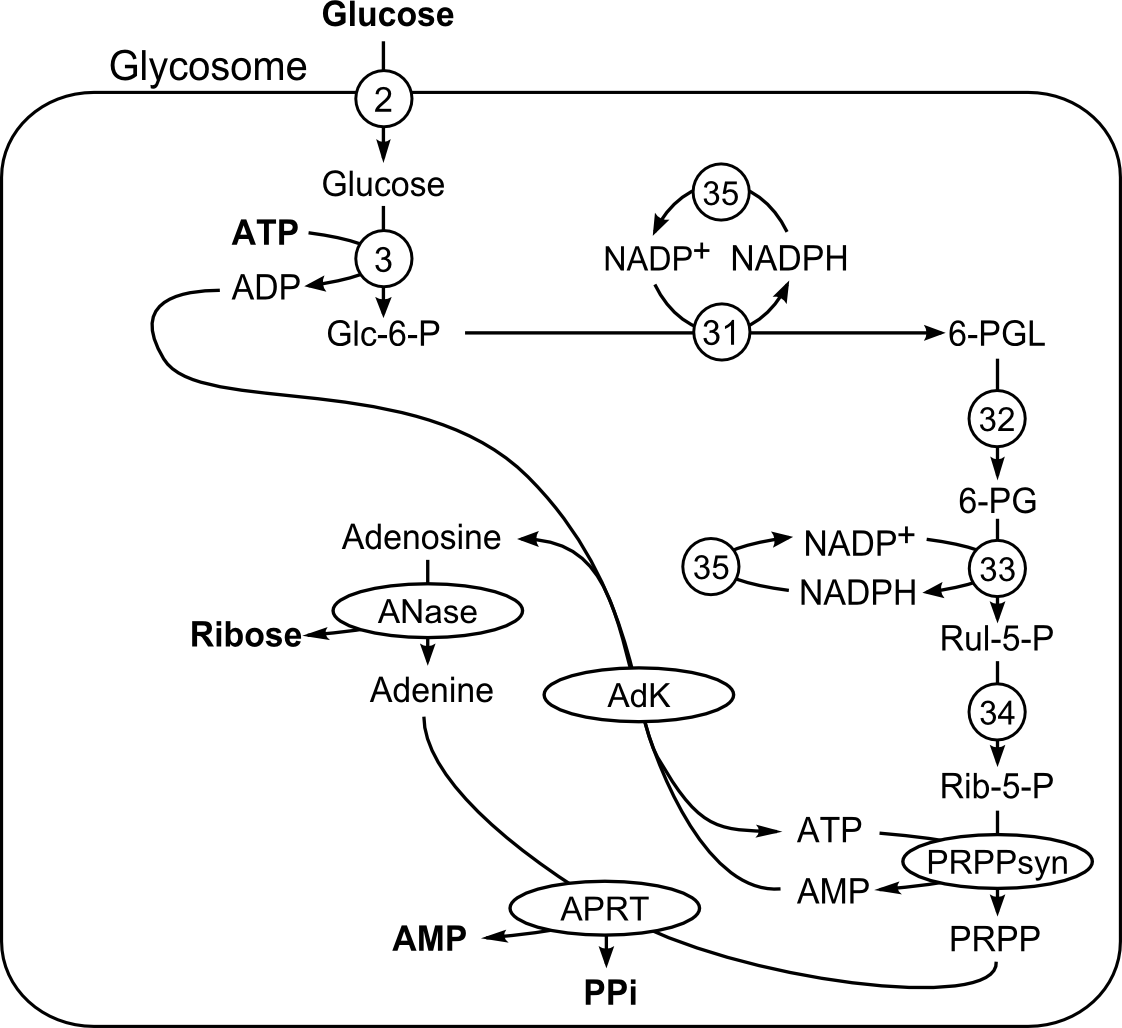

Supplement: Figure S1 — Enzymes of purine salvage pathway localized in glycosome. The glycosomal PPP is shown with those enzymes from the purine salvage pathway that have a predicted glycosomal localization (Table S3). Reactions from the PPP model are indicated by their number from Table 1, while the additional reactions are indicated as: PRPP: phosphoribosyl pyrophosphate synthetase; AdK: adenosine kinase; ANase: adenosine nucleosidase; APRT: adenine phosphoribosyltransferase. Metabolites that are not balanced within this pathway are indicated in bold. The scheme demonstrates how the presence of the purine salvage pathway is unable to rescue the phosphate leak, as the resulting overall reaction is glucose+ATP→ribose+AMP+PPi (+CO2, implied to be balanced with gaseous CO2). Additional ADK and ANase reactions does not improve this situation, with a resulting overall reaction of glucose+ADP→2 ribose+adenine+2 PPi (+CO2). In contrast, ribokinase is capable of resolving the phosphate leak with a resulting overall reaction of glucose→ribose (+CO2). (TIF) [file pcbi.1003371.s004.tif]

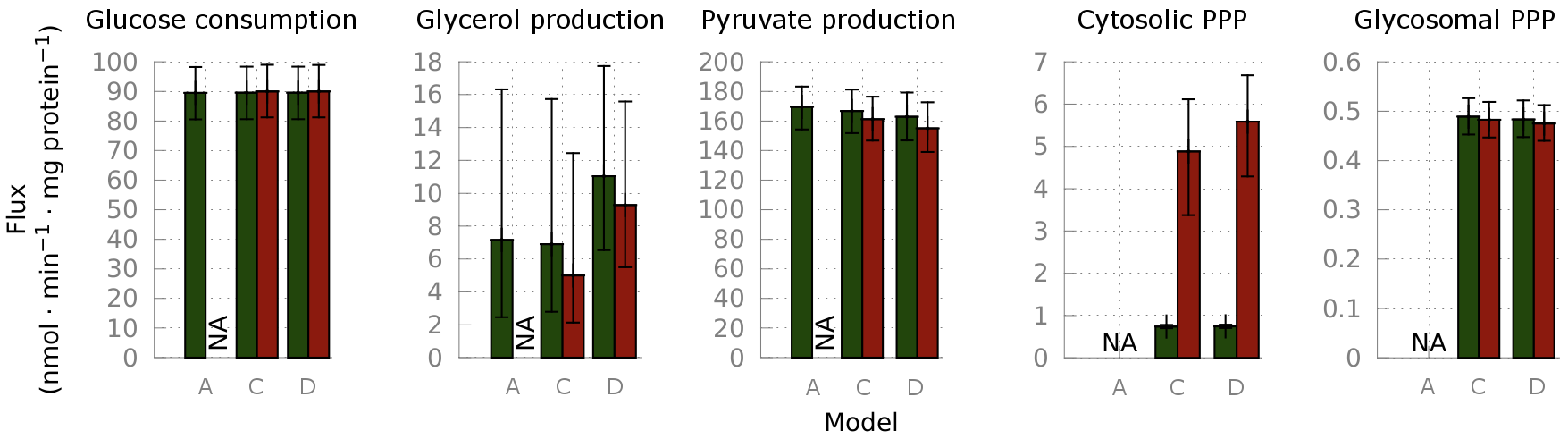

Supplement: Figure S2 — Steady-state fluxes through various models. Steady state fluxes of at standard conditions (green, kTOX = 2 µl·min−1·mg protein−1) and if cytosolic PPP is maximized (red, kTOX = 200 µl·min−1·mg protein−1). Error bars indicate interquartile ranges. NA denotes ‘Not Applicable’ for branches that are absent from certain model version, Glce is 5 mM in all models. The glucose consumption flux is distributed over the production of glycerol and pyruvate and the two branches of the PPP. Note that ALD generates two trioses from every hexose, such that the fluxes through the trioses glycerol and pyruvate are double the hexose flux. The errors bars indicated interquartile ranges as a result of the uncertainty modelling. The large error bars for glycerol production are a result of its low flux and the smaller uncertainties assigned to the fluxes through the other pathways. No information is given for model B, as this model is incomplete and cannot reach a steady state (outlined in the main text). (TIF) [file pcbi.1003371.s005.tif]

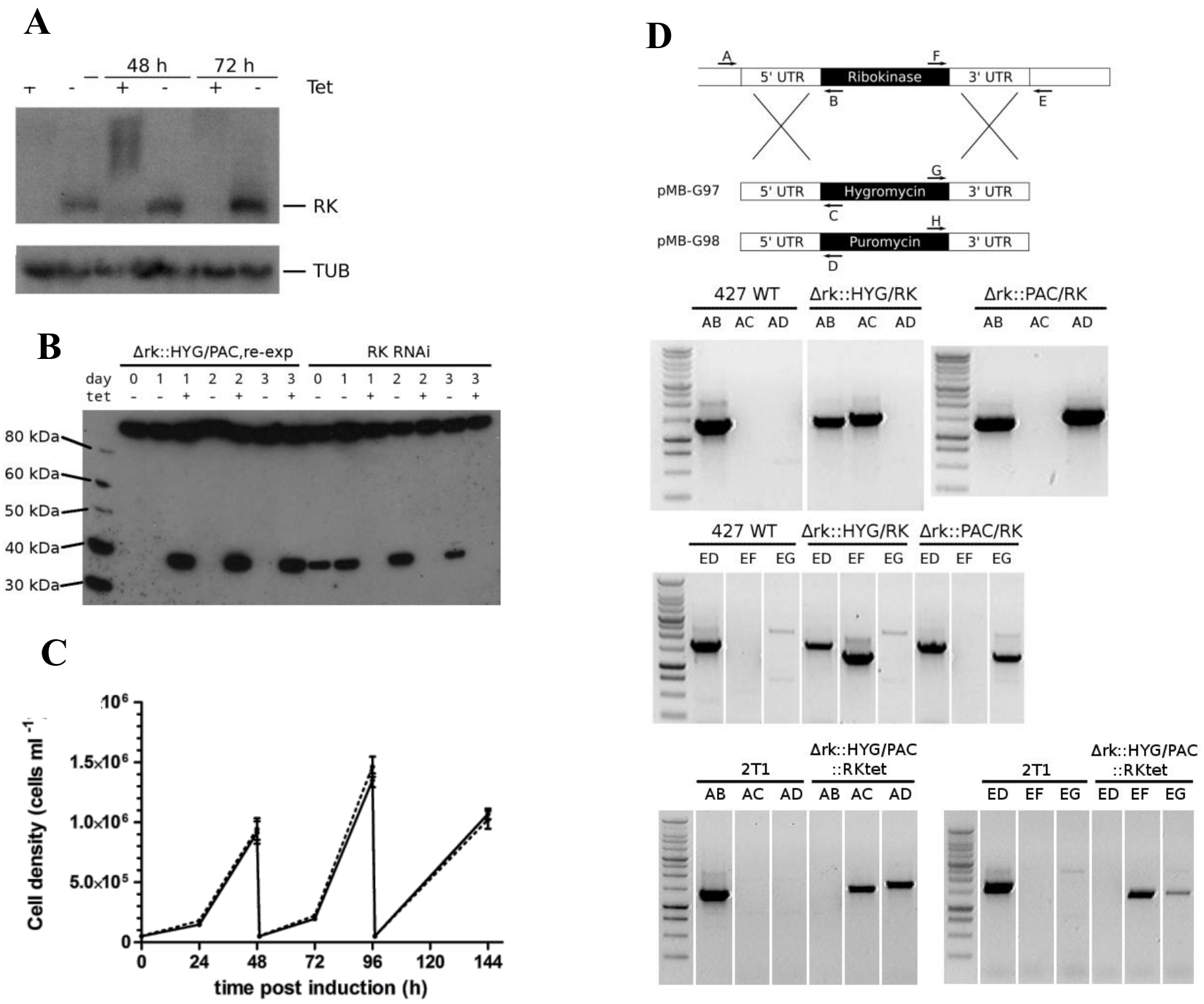

Supplement: Figure S3 — Genetic investigations in T. brucei ribokinase. (A) Northern blot of RKRNAi induced and non-induced T. brucei, samples taken 24, 48 and 72 hours post induction by tetracycline. Tubulin was used as loading control. (B) Western blot of RKRNAi and Δrk::HYG/PAC::RKtet, samples taken at t = 0, 1, 2 and 3 days post induction by tetracycline. (C) Growth curve of ribokinase knockdown mutant by RNA interference. Induction of RNAi was started at t = 0 by the addition of 1 µg/mL tetracycline. Cell densities of induced (dotted line) and control (solid line) cultures were determined by cell counts, and cultures were diluted down to 2·104 cells ml−1 at t = 48 and 96 h. No difference in growth effect could be observed. (D) Successful transfection of T. brucei with knockout constructs was confirmed by PCR. (TIF) [file pcbi.1003371.s006.tif]

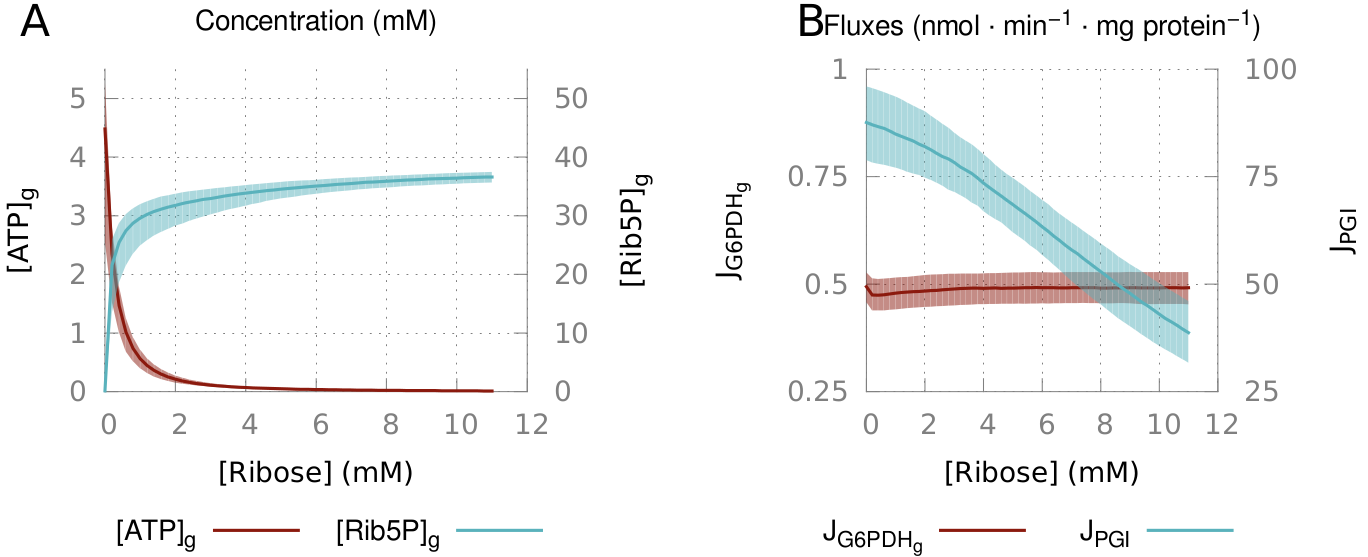

Supplement: Figure S4 — Ribose sensitivity. Steady state concentrations (A) and fluxes (B) of model C at various concentrations of ribose. Solid lines indicate medians, shaded areas show interquartile ranges. Increasing ribose concentration results in a depletion of ATP and accumulation of ribose 5-phosphate in the glycosome. While the glycosomal PPP flux (JG6PDHg) remains mostly unaffected, the glycolytic flux (JPGI) is strongly reduced. (TIF) [file pcbi.1003371.s007.tif]

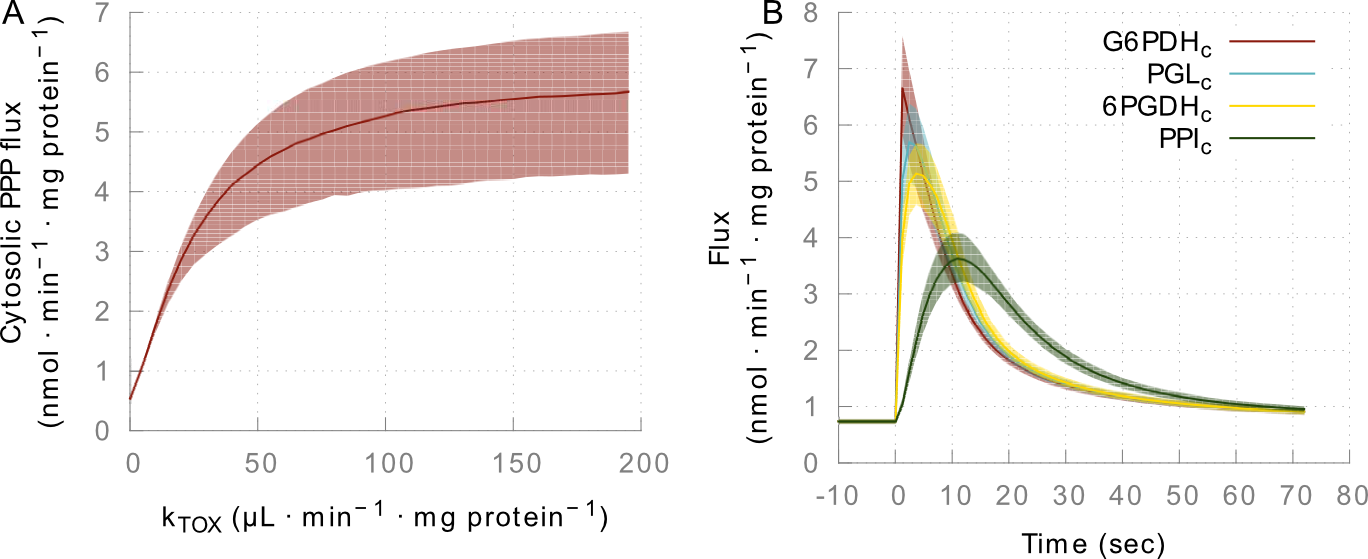

Supplement: Figure S5 — Simulations of oxidative stress in model D. Near identical to Figure 4, with results of model D instead. (A) The steady state flux through the cytosolic pentose phosphate pathway in model D as a function of the oxidative stress by varying the kinetic constant kTOX. (B) Fluxes through the cytosolic PPP enzymes as a function of time upon sudden oxidative stress. During the whole time-course, kTOX = 2 µl·min−1·mg protein−1. The system is removed from steady state at t = 0, by setting 99% of the NAD(P)H and trypanothione pools to the oxidized form. Shown is the relaxation of the cytosolic PPP fluxes. Solid lines indicate medians, shaded areas show interquartile ranges. (TIF) [file pcbi.1003371.s008.tif]

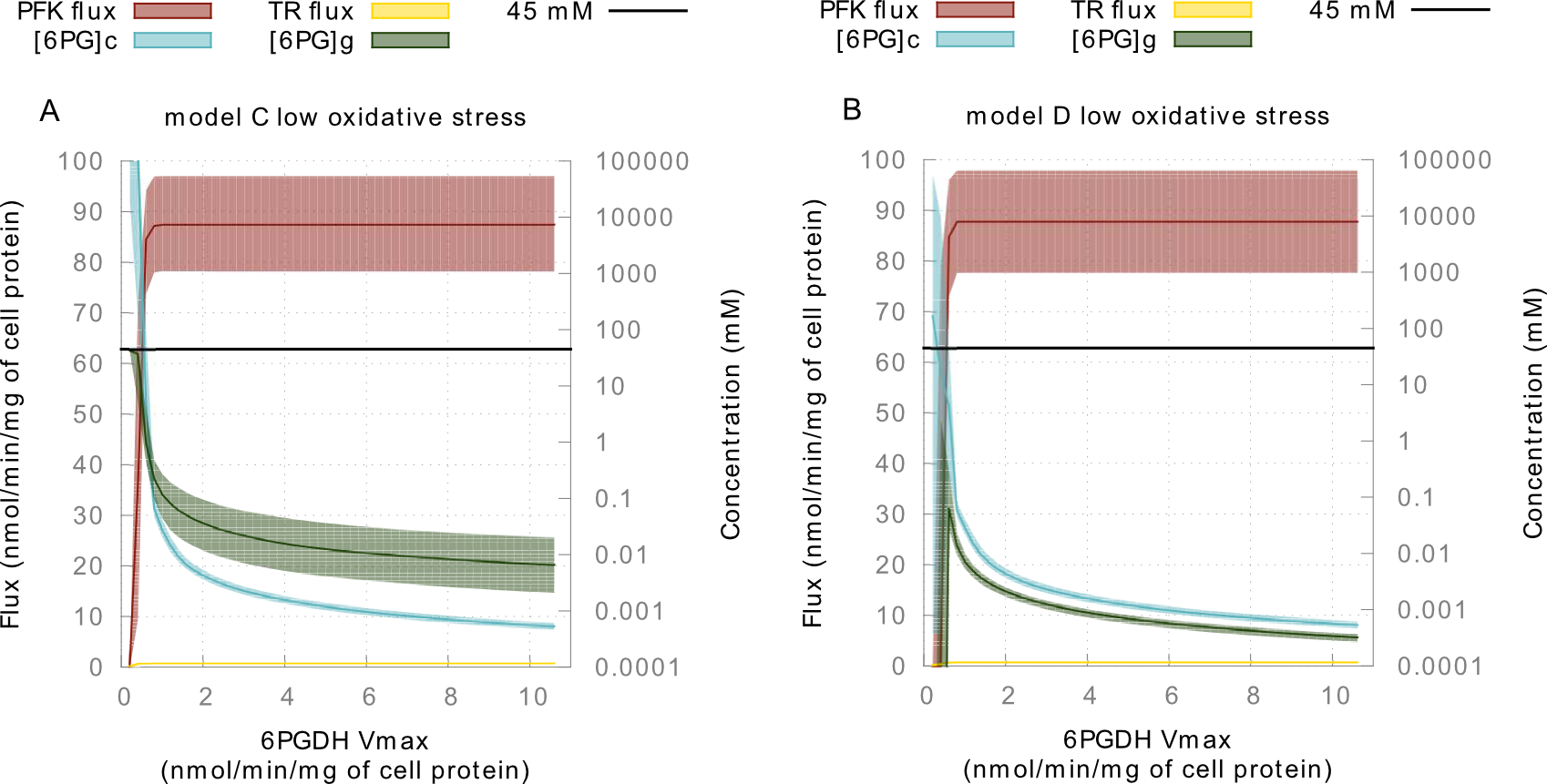

Supplement: Figure S6 — Simulations of 6PGDH inhibition and 6-PG accumulation. (A–B) The effects of inhibition of 6PGDH on 6-PG concentrations and metabolic fluxes were simulated by reducing V max,6PGDH in model C and D at low oxidative stress (kTOX = 2 µl·min−1·mg protein−1). Results for simulations at low oxidative stress (kTOX = 200 µl·min−1·mg protein−1) are shown in Figure 5. ATP production flux as steady-state flux through PFK is indicated in red, while trypanothione reductase steady-state flux is indicated in yellow, both plotted on the left y-axis. Steady-state concentration of cytosolic (blue) and glycosomal (green) 6-phosphogluconate are plotted on the right y-axis. Shaded areas indicate interquartile ranges. (TIF) [file pcbi.1003371.s009.tif]

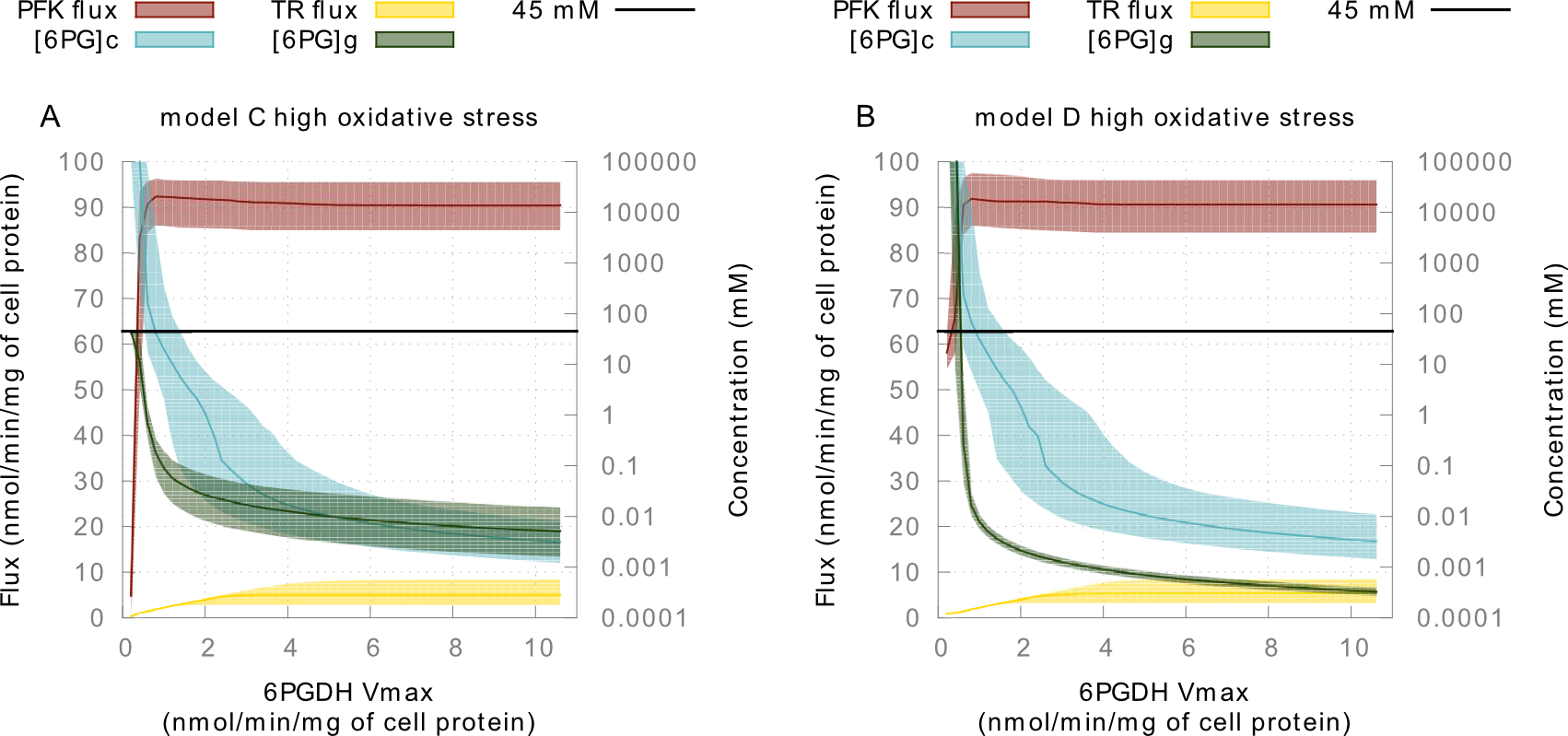

Supplement: Figure S7 — Simulations of 6PGDH inhibition during growth on fructose. (A–B) The effects of inhibition of 6PGDH on 6-PG concentrations and metabolic fluxes were simulated by reducing V max,6PGDH in model C and D at high oxidative stress (kTOX = 200 µl·min−1·mg protein−1), similar to Figure 5. ATP production flux as steady-state flux through PFK is indicated in red, while trypanothione reductase steady-state flux is indicated in yellow, both plotted on the left y-axis. Steady-state concentration of cytosolic (blue) and glycosomal (green) 6-phosphogluconate are plotted on the right y-axis. Shaded areas indicate interquartile ranges. (TIF) [file pcbi.1003371.s010.tif]

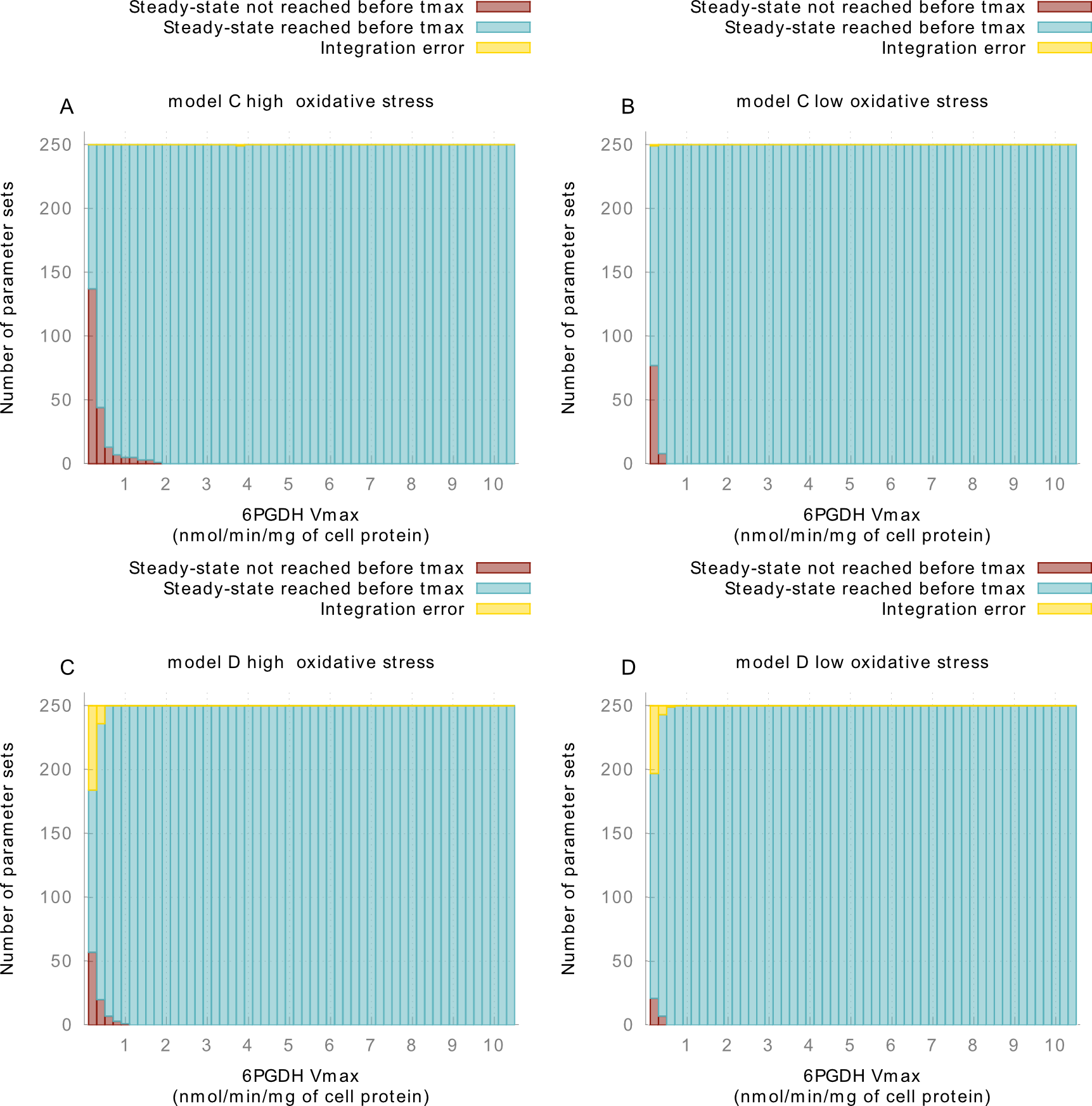

Supplement: Figure S8 — Percentage of models reaching steady-state within 10 million simulation minutes during 6PGDH inhibitions. As explained in detail in Text S1, 250 random parameter sets were used for calculating steady-states during 6PGDH inhibition (Figures 5 and S5). Outcome of model C are shown at high (A) and low (B) oxidative stress; and in model D at high (C) and low (D) oxidative stress. Panel A and C correspond to Figures 5A–B, while panel B and D correspond to Figures S6A–B. (TIF) [file pcbi.1003371.s011.tif]

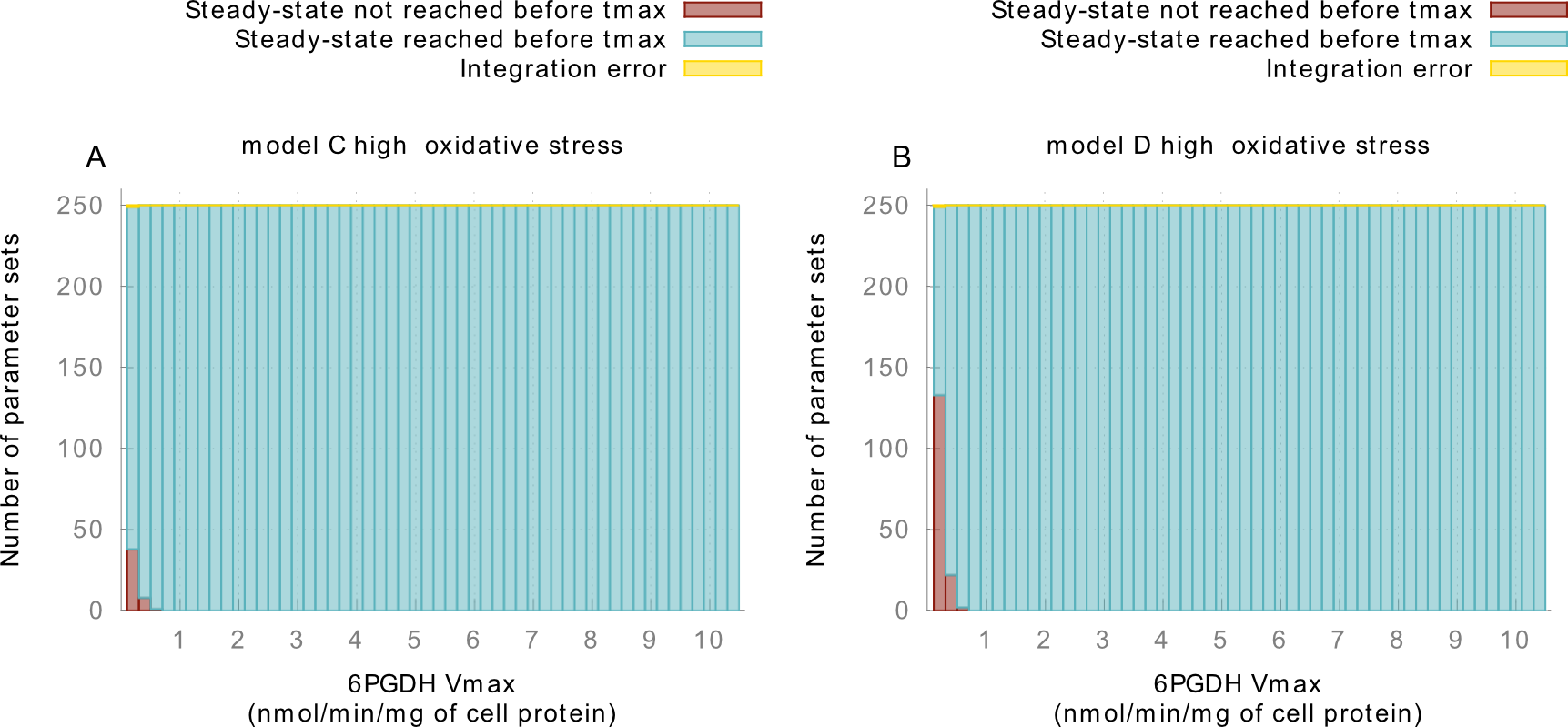

Supplement: Figure S9 — Percentage of models reaching steady-state within 10 million simulation minutes during 6PGDH inhibitions with growth on fructose. As explained in detail in Text S1, 250 random parameter sets were used for calculating steady-states during 6PGDH inhibition (Figures 6 and S6). Outcome at high oxidative stress are shown for model C (A) and D (B). Results from the simulations are shown in Figure S7. (TIF) [file pcbi.1003371.s012.tif]
