# Supplementary material for: Handling Uncertainty in Dynamic Models: The Pentose Phosphate Pathway in Trypanosoma brucei
Source: PLoS Comput Biol. 2013 Dec 5;9(12):e1003371. doi: 10.1371/journal.pcbi.1003371 (PMC3854711; doi:10.1371/journal.pcbi.1003371)
Supplement: Table S1 — Kinetic parameters of the enzymes related to the PPP. Activities, which depend on the expression level of the enzymes, are specified separately for the fraction of the enzyme that is localized in the glycosome and, if applicable, for the fraction which is located in the cytosol. c/g indicates that cytosolic and glycosomal activities are identical. Other parameters are assumed to be identical for glycosomal and cytosolic enzyme fractions. Parameter values given here are used in the fixed parameter models. Distributions of parameter values used in uncertainty modelling are given in Text S1. (DOCX) [file pcbi.1003371.s013.docx]

| **Enzyme** | **Parameter** | **Value** | **Unit** | **Ref.** |
| --- | --- | --- | --- | --- |
| G6PDH | *V_max,c/g_* | 8.4 | nmol · min^−1^ · mg protein^−1^ | [1,2] |
|  | *K_eq_* | 5.02 | dimensionless | [3]*^a^* |
|  | *K_m,Glc-6-P_* | 0.058 | mM | [4] |
|  | *K_m, 6-PGL_* | 0.04 | mM | [5]*^b^* |
|  | *K_m, NADP+_* | 0.0094 | mM | [4] |
|  | *K_m, NADPH_* | 0.0001 | mM | [5]*^b^* |
| PGL | *V_max,g_* | 5 | nmol · min^−1^ · mg protein^−1^ | [1,6] |
|  | *V_max,c_* | 28 | nmol · min^−1^ · mg protein^−1^ | [1,6] |
|  | *K_eq_* | 20000 | dimensionless | [7]*^a^* |
|  | *K_m,6-PGL_* | 0.05 | mM | [8]*^c^* |
|  | *K_m,6-PG_* | 0.05 | mM | *d* |
|  | *k* | 0.055 | min^−1^ | [9] |
| 6PGDH | *V_max,c/g_* | 10.6 *^e^* | nmol · min^−1^ · mg protein^−1^ | [1] |
|  | *K_eq_* | 47 | dimensionless | [10] |
|  | *K_m,6-PG_* | 0.0035 | mM | [11] |
|  | *K_m,Rul-5-P_* | 0.03 | mM | [11] |
|  | *K_m,NADP+_* | 0.001 | mM | [11] |
|  | *K_m,NADPH_* | 0.0006 | mM | [11] |
| PPI | *V_max,c/g_* | 72 *^e^* | nmol · min^−1^ · mg protein^−1^ | [1] |
|  | *K_eq_* | 5.6 | dimensionless | [12] |
|  | *K_m,Rul5P_* | 4 | mM | [8]*^c^* |
|  | *K_m,Rib5P_* | 1.4 | mM | [8]*^c^* |
| TR | *V_max_* | 252 | nmol · min^−1^ · mg protein^−1^ | [13]*^f^* |
|  | *K_eq_* | 434 | dimensionless | [14]*^g^* |
|  | *K_m,TS2_* | 0.0069 | mM | [15] |
|  | *K_m,TSH2_* | 0.0018 | mM | *h* |
|  | *K_m,NADPH_* | 0.00077 | mM | [15] |
|  | *K_m,NADP_* | 0.081 | mM | *h* |
| TOX | *k* | 2-200 | μl · min^−1^ · mg protein^−1^ | Varies depending on oxidative stress |
| NADPHu | *k_c/g_* | 2 | μl · min^−1^ · mg protein^−1^ | *i* |
| G6PP | *V_max_* | 21 | nmol · min^−1^ · mg protein^−1^ | This paper |
|  | *K_eq_* | 263 | dimensionless | [16] |
|  | *K_m,Glc-6-P_* | 5.6 | mM | [17]*^j^* |
|  | *K_m,Glc_* | 5.6 | mM | [17]*^d,j^* |
| ATP:ADP | *V_max_* | 1.5 | nmol · min^−1^ · mg protein^−1^ | *k* |
| antiporter | *K_eq_* | 1 | dimensionless | *k* |
|  | *K_m,ATPc/g_* | 0.02 | mM | *k* |
|  | *K_m,ADPc/g_* | 0.02 | mM | *k* |
| RK | *V_max,g_* | 5 | nmol · min^−1^ · mg protein^−1^ | *l* |
|  | *K_eq_* | 0.0036 | dimensionless | [18] |
|  | *K_m,Rib-5-P_* | 0.39 | mM | This paper |
|  | *K_m,Rib_* | 0.51 | mM | This paper |
|  | *K_m,ADP_* | 0.25 | mM | This paper |
|  | *K_m,ATP_* | 0.24 | mM | This paper |
| FruT | *V_max_* | 69.1 | nmol · min^−1^ · mg protein^−1^ | [19] |
|  | *K_m,Fru_* | 3.91 | mM | [19] |
|  | α | 0.75 | dimensionless | *m* |
| HXK(Fru) | *V_max,c_* | 1775 | nmol · min^−1^ · mg protein^−1^ | *n* |
|  | *V_max,g_* | 154 | nmol · min^−1^ · mg protein^−1^ | *n* |
|  | *K_eq_* | 631 | dimensionless | [20] |
|  | *K_m,Fru_* | 0.35 | mM | [21] |
|  | *K_m,Fru-6-P_* | 12 | mM | *n* |
|  | *K_m,ATP_* | 0.116 | mM | *n* |
|  | *K_m,ADP_* | 0.126 | mM | *n* |
|  | *K_i,Glc_* | 0.1 | mM | *o* |
|  | *K_i,Glc-6-P_* | 12 | mM | *o* |
| HXK(Glc) | *K_i,Fru_* | 0.35 | mM | *o* |
|  | *K_i,Fru-6-P_* | 12 | mM | *o* |

*a:* Corrected to pH 7 and/or 25 ºC*.*

*b:* Parameter derived from human red blood cells. *K_m_* values for Glc-6-P and NADP^+^ are in the same range for *T. brucei* and RBCs. Calculations can be found on SilicoTryp wiki (http://silicotryp.ibls.gla.ac.uk/wiki/).

*c:* Parameter derived from *Trypanosoma cruzi*.

*d:* No value available. The affinity is assumed to be similar for the substrate and the product.

*e:* No reliable localization data available, assumed to be distributed equally in glycosome and cytosol.

*f:* Corrected from activity per number of cells to activity per mg cell protein by using 1.94 · 10^8^ cells · mg cell protein^−1^, as used by [22].

*g:* Calculated from redox potential of trypanothione.

*h:* Ratio of *K_m_* values of reduced and oxidized trypanothione are assumed to be similar to the ratio of *K_m_* values of reduced and oxidized glutathione for glutathione reductase.

*i:* Fitted to result in a total PPP flux of 1.2 nmol · min^−1^ · mg protein^−1^, which is the average PPP flux in *T. cruzi* and *L. mexicana* without induced oxidative stress [23,24]. The PPP flux is assumed to be distributed equally between cytosol and glycosome when no additional oxidative stress is present (*k_TOX_* = 0).

*j:* The average of the two types of acid phosphatases in *T. rhodesiense* were used as a first estimate.

*k:* The hypothetical ATP:ADP antiporter was given arbitrary kinetic constants. The *K_eq_* of 1 reflects the assumption of facilitated transport, i.e. not driven by an external source of Gibbs free energy.

*l:* Attempts to measure the intracellular ribokinase activity in cellular lysates were unsuccessful, as other enzymes present in the cell extracts confounded the results. The ribokinase *V_max_*-value was therefore sampled from a wide range.

*m:* No value available, assumed to be similar to glucose transporter.

*n:* No value measured for fructose (6-phosphate). Assumed to be similar to the value for glucose (6-phosphate).

*o:* Competitive inhibitor, K_i_ is identical to K_m_.

1. Cronin CN, Nolan DP, Paul Voorheis H (1989) The enzymes of the classical pentose phosphate pathway display differential activities in procyclic and bloodstream forms of Trypanosoma brucei. FEBS Lett 244: 26–30. doi:10.1016/0014-5793(89)81154-8.

2. Heise N, Opperdoes FR (1999) Purification, localisation and characterisation of glucose-6-phosphate dehydrogenase of Trypanosoma brucei. Mol Biochem Parasitol 99: 21–32.

3. Goldberg RN, Tewari YB, Bell D, Fazio K, Anderson E (1993) Thermodynamics of Enzyme-Catalyzed Reactions: Part 1. Oxidoreductases. J Phys Chem Ref Data 22: 515–582.

4. Cordeiro AT, Thiemann OH, Michels PAM (2009) Inhibition of Trypanosoma brucei glucose-6-phosphate dehydrogenase by human steroids and their effects on the viability of cultured parasites. Bioorg Med Chem 17: 2483–2489. doi:doi: DOI: 10.1016/j.bmc.2009.01.068.

5. Thorburn DR, Kuchel PW (1985) Regulation of the human-erythrocyte hexose-monophosphate shunt under conditions of oxidative stress. A study using NMR spectroscopy, a kinetic isotope effect, a reconstituted system and computer simulation. Eur J Biochem FEBS 150: 371–386.

6. Duffieux F, Van Roy J, Michels PA, Opperdoes FR (2000) Molecular characterization of the first two enzymes of the pentose-phosphate pathway of Trypanosoma brucei. Glucose-6-phosphate dehydrogenase and 6-phosphogluconolactonase. J Biol Chem 275: 27559–27565. doi:10.1074/jbc.M004266200.

7. Casazza JP, Veech RL (1986) The interdependence of glycolytic and pentose cycle intermediates in ad libitum fed rats. J Biol Chem 261: 690–698.

8. Igoillo-Esteve M, Maugeri D, Stern AL, Beluardi P, Cazzulo JJ (2007) The pentose phosphate pathway in Trypanosoma cruzi: a potential target for the chemotherapy of Chagas disease. An Acad Bras Ciências 79: 649–663.

9. Schofield PJ, Sols A (1976) Rat liver 6-phosphogluconolactonase: a low Km enzyme. Biochem Biophys Res Commun 71: 1313–1318.

10. Villet RH, Dalziel K (1969) The nature of the carbon dioxide substrate and equilibrium constant of the 6-phosphogluconate dehydrogenase reaction. Biochem J 115: 633–638.

11. Hanau S, Rippa M, Bertelli M, Dallocchio F, Barrett MP (1996) 6-Phosphogluconate dehydrogenase from Trypanosoma brucei. Eur J Biochem 240: 592–599. doi:10.1111/j.1432-1033.1996.0592h.x.

12. Goldberg RN, Tewari YB (1995) Thermodynamics of Enzyme-Catalyzed Reactions: Part 5. Isomerases and Ligases. J Phys Chem Ref Data 24: 1765–1801.

13. Krieger S, Schwarz W, Ariyanayagam MR, Fairlamb AH, Krauth-Siegel RL, et al. (2000) Trypanosomes lacking trypanothione reductase are avirulent and show increased sensitivity to oxidative stress. Mol Microbiol 35: 542–552.

14. Fairlamb AH, Cerami A (1992) Metabolism and functions of trypanothione in the Kinetoplastida. Annu Rev Microbiol 46: 695–729. doi:10.1146/annurev.mi.46.100192.003403.

15. Jones DC, Ariza A, Chow W-HA, Oza SL, Fairlamb AH (2010) Comparative structural, kinetic and inhibitor studies of Trypanosoma brucei trypanothione reductase with T. cruzi. Mol Biochem Parasitol 169: 12–19. doi:10.1016/j.molbiopara.2009.09.002.

16. Atkinson MR, Johnson E, Morton RK (1961) Equilibrium constants of phosphoryl transfer from C-1 to C-6 of alpha-D-glucose 1-phosphate and from glucose 6-phosphate to water. Biochem J 79: 12–15.

17. McLaughlin J (1986) The association of distinct acid phosphatases with the flagella pocket and surface membrane fractions obtained from bloodstream forms of Trypanosoma rhodesiense. Mol Cell Biochem 70: 177–184.

18. Tewari YB, Steckler DK, Goldberg RN, Gitomer WL (1988) Thermodynamics of hydrolysis of sugar phosphates. J Biol Chem 263: 3670–3675.

19. Fry AJ, Towner P, Holman GD, Eisenthal R (1993) Transport of d-fructose and its analogues by Trypanosoma brucei. Mol Biochem Parasitol 60: 9–18. doi:10.1016/0166-6851(93)90023-Q.

20. Banks BE, Vernon CA (1970) Reassessment of the role of ATP in vivo. J Theor Biol 29: 301–326.

21. Morris MT, DeBruin C, Yang Z, Chambers JW, Smith KS, et al. (2006) Activity of a Second Trypanosoma brucei Hexokinase Is Controlled by an 18-Amino-Acid C-Terminal Tail. Eukaryot Cell 5: 2014–2023. doi:10.1128/EC.00146-06.

22. Bakker BM, Michels PAM, Opperdoes FR, Westerhoff HV (1999) What controls glycolysis in bloodstream form Trypanosoma brucei? J Biol Chem 274: 14551–14559. doi:10.1074/jbc.274.21.14551.

23. Maugeri DA, Cazzulo JJ, Burchmore RJS, Barrett MP, Ogbunude POJ (2003) Pentose phosphate metabolism in Leishmania mexicana. Mol Biochem Parasitol 130: 117–125.

24. Maugeri DA, Cazzulo JJ (2004) The pentose phosphate pathway in Trypanosoma cruzi. FEMS Microbiol Lett 234: 117–123. doi:10.1016/j.femsle.2004.03.018.
