# Supplementary material for: Handling Uncertainty in Dynamic Models: The Pentose Phosphate Pathway in Trypanosoma brucei
Source: PLoS Comput Biol. 2013 Dec 5;9(12):e1003371. doi: 10.1371/journal.pcbi.1003371 (PMC3854711; doi:10.1371/journal.pcbi.1003371)
Supplement: Table S2 — Elementary flux modes in models of PPP. The elementary modes of different model versions including the glycosomal PPP are listed as the overall reactions plus in brackets the individual enzyme-catalyzed reactions with their relative flux weight. For simplicity the cytosolic PPP and the cytosolic NADPH utilization were left out of this analysis. The modules included in each model version refer to Table 1 and the color-coded extensions in Figure 1 in the main text. A negative number indicates that the reaction occurs in the reverse direction as compared to Table 1 in the main text. The order of reactions corresponds to that in Table 1 in the main text. The glycolytic modes 1–3 are possible in all model versions, but are not listed again for the model versions extended with the glycosomal PPP. Elementary mode analysis of model B only results in the flux modes 1–3. (DOCX) [file pcbi.1003371.s014.docx]

| **Glycolysis** | |
| --- | --- |
| 1. | Aerobic glycolysis  Glc*_out_* + O_2_ →2 Pyr*_out_*  (GlcT*_plasmamembrane_*, GlcT*_glycosomal membrane_*, HXK*_g_*, PGI, PFK, ALD, TPI, 2 GAPDH, 2 PGK, 2 PGAT, 2 PGAM, 2 ENO, 2 PYK, 2 PyrT, 2 GDH, 2 DHAP:Gly-3-P antiporter, 2 GPO, 2 ATP utilization) |
| 2. | Anaerobic glycolysis  Glc*_out_* →Pyr*_out_* + Gly*_out_*  (GlcT*_plasmamembrane_*, GlcT*_glycosomal membrane_*, HXK*_g_*, PGI, PFK, ALD, GAPDH, PGK, PGAT, PGAM, ENO, PYK, PyrT, GDH, GK, ATP utilization) |
| 3. | Glycerol oxidation  Gly*_out_* + O_2_ → Pyr*_out_*  (TPI, GAPDH, PGK, PGAT, PGAM, ENO, PYK, PyrT, GDH, 2 DHAP:Gly-3-P antiporter, 2 GPO, ‑GK, ATP utilization) |
| **Glycolysis + glycosomal PPP + Ribokinase** | |
| Modes 1–3 plus: | |
| 4. | Glycosomal PPP  Glc*_out_* → Rib*_out_* + CO_2_  (GlcT*_plasmamembrane_*, GlcT*_glycosomal membrane_*, HXK*_g_*, G6PDH, PGL, 6PGDH, PPI, 2 NADPH oxidation, RK) |
| **Glycolysis + glycosomal PPP + ATP transport** | |
| Modes 1–3 plus: | |
| 5. | Aerobic glycolysis plus PPP  3 Glc*_out_* + O_2_ → 2 Pyr*_out_* + 2 Rib-5-P*_g_* +CO_2_  (3 GlcT*_plasmamembrane_*, 3 GlcT*_glycosomal membrane_*, 3 HXK*_g_*, PGI, PFK, ALD, TPI, 2 GAPDH, 2 PGK, 2 PGAT, 2 PGAM, 2 ENO, 2 PYK, 2 PyrT, 2 GDH, 2 DHAP-Gly-3-P antiporter, 2 GPO, 2 G6PDH, 2 PGL, 2 6PGDH, 2 PPI, 4 NADPH oxidation, 2 ATP:ADP antiporter) |
| 6. | Anaerobic glycolysis plus PPP  2 Glc*_out_* → Pyr*_out_* + Gly*_out_* + Rib-5-P*_g_* + CO_2_  (2 GlcT*_plasmamembrane_*, 2 GlcT*_glycosomal membrane_*, 2 HXK*_g_*, PGI, PFK, ALD, GAPDH, PGK, PGAT PGAM, ENO, PYK, PyrT, GDH, GK, G6PDH, PGL, 6PGDH, PPI, 2 NADPH oxidation, ATP:ADP antiporter) |
| 7. | Glycerol oxidation plus PPP  Gly*_out_* + O_2_ + Glc*_out_* → Pyr*_out_* + Rib-5-P*_g_* + CO_2_  (GlcT*_plasmamembrane_*, GlcT*_glycosomal membrane_*, HXK, TPI, GAPDH, PGK, PGAT, PGAM, ENO, PYK, PyrT, GDH, 2 DHAP:Gly-3-P antiporter, 2 GPO, -GK, G6PDH, PGL, 6PGDH, PPI, 2 NADPH oxidation, ATP:ADP antiporter) |
