# Supplementary material for: Handling Uncertainty in Dynamic Models: The Pentose Phosphate Pathway in Trypanosoma brucei
Source: PLoS Comput Biol. 2013 Dec 5;9(12):e1003371. doi: 10.1371/journal.pcbi.1003371 (PMC3854711; doi:10.1371/journal.pcbi.1003371)
Supplement: Table S3 — Predicted glycosomal proteome. All reactions present in the bloodstream form T. brucei glycosome, according to comprehensible glycosomal proteomics [18], [19]. Only reactions present in the bloodstream form of the parasite are included. Abbreviations and EC numbers are given for each reaction. Models are indicated where reactions are part of a model in the main text. Indicated are what reactions are part of an elementary model (see Table S4). (DOCX) [file pcbi.1003371.s015.docx]

| **Abbreviation** | **EC** | **Enzyme name** | **Model** | **Elementary mode** | **Ref.** |
| --- | --- | --- | --- | --- | --- |
| OGDH | 1.2.4.2 | 2-oxoglutarate dehydrogenase |  |  | [1] |
| SRD5A | 1.3.99.5 | 3-oxo-5-alpha-steroid 4-dehydrogenase |  |  | [1] |
| 6PGDH | 1.1.1.43 | 6-phosphogluconate dehydrogenase | B, C, D | × | [2] |
| APRT | 2.4.2.7 | Adenine phosphoribosyltransferase |  | × | [2] |
| AdK | 2.7.1.20 | Adenosine kinase |  | × | [2] |
| ANase | 3.2.2.7 | Adenosine nucleosidase |  | × | [2] |
| AK | 2.7.4.3 | Adenylate kinase | A – D | × | [1] |
| AdSS | 6.3.4.4 | Adenylosuccinate synthetase |  |  | [2] |
| ALD | 4.1.2.13 | Aldolase | A – D | × | [1,2] |
| AGPS | 2.5.1.26 | Alkyl-dihydroxyacetone phosphate synthase |  |  | [1] |
| ArgK | 2.7.3.3 | Arginine kinase |  |  | [1,2] |
| FBPase | 3.1.3.11 | Fructose bisphosphatase |  | × | [1] |
| FRD | 1.3.1.6 | Fumarate reductase |  |  | [2] |
| G6PDH | 1.1.1.49 | Glucose-6-phosphate dehydrogenase | A – D | × | [2] |
| GAPDH | 1.2.1.12 | Glyceraldehyde-3-phosphate dehydrogenase | A – D | × | [1,2] |
| GK | 2.7.1.30 | Glycerol kinase | A – D | × | [1,2] |
| G3PDH | 1.1.1.8 | Glycerol-3-phosphate dehydrogenase | A – D | × | [1,2] |
| GPO | 1.1.3.21 | Glycerol-3-phosphate oxidase |  | × | [1,2] |
| GDA | 3.5.4.15 | Guanine deaminase |  |  | [2] |
| HGPRT | 2.4.2.8 | Guanine phosphoribosyltransferase |  |  | [2] |
| GNase | 3.2.2.1 | Guanosine nucleosidase |  |  | [2] |
| HXK | 2.7.1.1 | Hexokinase | A – D | × | [1,2] |
| HPRT | 2.4.2.8 | Hypoxanthine phosphoribosyltransferase |  |  | [1] |
| IRH | 3.2.2.2 | Inosine nucleosidase |  |  | [2] |
| IMPDH | 1.1.1.205 | Inosine-5'-monophosphate dehydrogenase |  |  | [1,2] |
| SOD | 1.15.1.1 | Iron superoxide dismutase |  |  | [2] |
| IDH | 1.1.1.41 | Isocitrate dehydrogenase |  |  | [1,2] |
| TDH | 1.1.1.103 | L-Threonine 3-dehydrogenase |  |  | [1] |
| LysoPLA | 3.1.1.5 | Lysophospholipase |  |  | [1] |
| MVK | 2.7.1.36 | Mevalonate kinase |  |  | [2] |
| NADPHu |  | NADPH utilization | B, C, D | × |  |
| OMPRT | 2.4.2.10 | Orotate phosphoribosyltransferase |  |  | [1,2] |
| OMPDC | 4.1.1.23 | Orotidine-5-phosphate decarboxylase |  |  | [1,2] |
| PPI | 5.3.1.6 | Pentosephosphate isomerase | B, C, D | × | [2] |
| PEPCK | 4.1.1.32 | Phosphoenolpyruvate carboxykinase |  |  | [2] |
| PFK | 2.7.1.11 | Phosphofructokinase | A – D | × | [1,2] |
| PGL | 3.1.1.31 | Phosphogluconolactonase | B, C, D | × |  |
| PGI | 5.3.1.9 | Phosphoglucose isomerase | A – D | × | [1,2] |
| PGK | 2.7.2.3 | Phosphoglycerate kinase | A – D | × | [1,2] |
| PMI | 5.3.1.8 | Phosphomannose isomerase |  |  | [2] |
| PRPPsyn | 2.7.6.1 | Phosphoribosyl pyrophosphate synthetase |  | × | [2] |
| PYK | 2.7.1.40 | Pyruvate kinase |  |  | [1] |
| RK | 2.7.1.15 | Ribokinase | C | × | [1,2] |
| TPI | 5.3.1.1 | Triosephosphate isomerase | A – D | × | [1,2] |
| GALE | 5.1.3.2 | UDP-galactose 4-epimerase |  |  | [2] |
| XNase | 3.2.2.1 | Xanthosine nucleosidase |  |  | [2] |

1. Colasante C, Ellis M, Ruppert T, Voncken F (2006) Comparative proteomics of glycosomes from bloodstream form and procyclic culture form Trypanosoma brucei brucei. Proteomics 6: 3275–3293. doi:10.1002/pmic.200500668.

2. Vertommen D, Van Roy J, Szikora J-P, Rider MH, Michels PAM, et al. (2008) Differential expression of glycosomal and mitochondrial proteins in the two major life-cycle stages of Trypanosoma brucei. Mol Biochem Parasitol 158: 189–201. doi:10.1016/j.molbiopara.2007.12.008.
