# Supplementary material for: Handling Uncertainty in Dynamic Models: The Pentose Phosphate Pathway in Trypanosoma brucei
Source: PLoS Comput Biol. 2013 Dec 5;9(12):e1003371. doi: 10.1371/journal.pcbi.1003371 (PMC3854711; doi:10.1371/journal.pcbi.1003371)
Supplement: Table S4 — Elementary modes in glycosomal proteome. A model of glycosomal metabolism was constructed for use in METATOOL version 4.9.3 [60]. The reactions from Table S3 were used. All reactions were set as reversible, except for alternative oxidase (TAO), phosphofructokinase (PFK), fructose bisphosphatase (FBPase), and phosphogluconolactonase (PGL). Protons, Pi, PPi, H2O, CO2 and O2 were not included in the reactions. Glucose, 3-phosphoglyceric acid, ribose and glycerol were set as external metabolites. The resulting model has 9 elementary modes, where the first four modes are also occurring in the models described in this paper. Elementary mode 5 is a futile cycle without external metabolites involved. Elementary modes 6–9 are unlikely to occur in dividing bloodstream trypanosomes, as a high glucose to pyruvate and glycerol flux is maintained. Additionally, the activity of fructose bisphosphatase could not be measured [23], [61]. (DOCX) [file pcbi.1003371.s016.docx]

| **Glycosomal elementary modes** | |
| --- | --- |
| 1. | Aerobic glycolysis  Glc → 2 3PGA  (HXK, PGI, PFK, ALD, TPI, 2 GAPDH, 2 PGK, 2 GDH, 2 GPO) |
| 2. | Anaerobic glycolysis  Glc → 3PGA + Gly  (HXK, PGI, PFK, ALD, GAPDH, PGK, GDH, GK) |
| 3. | Glycerol oxidation  Gly → 3PGA  (GK, 2 GPO, GDH, TPI, GAPDH, PGK) |
| 4. | Glycosomal PPP  Glc → Rib  (HXK, G6PDH, PGL, 6PGDH, PPI, 2 NADPHu, RK) |
| 5. | Futile cycle  No external metabolites involved  (2 PFK, 2FBPase, RK, AK, APRT, ANase, -PRPPsyn, ADK) |
| 6. | Gluconeogenesis from glycerol  4 Gly → 2 Glc  (4 -GK, 4 TAO, 2 TPI, 2 -ALD, 2 FBPase, 2 -PGI, 2 -HK, RK, AK, APRT, ANase, -PRPPsyn, ADK) |
| 7. | Riboneogenesis from glycerol  4 Gly → 2 Rib  (4 -GK, 4 TAO, 2 TPI, 2 -ALD, 2 FBPase, 2 -PGI, 2 G6PDH, 2 PGL, 2 6PGDH, 2 PPI, 3 RK, AK, APRT, ANase, -PRPPsyn, ADK, 4 NADPHu) |
| 8. | Gluconeogenesis from glycerol and 3PGA  2 3PGA + 2 Gly 🡪 2 Glc  (2 -PGK, 2 -GAPDH, 2 -GK, 2 -GDH, 2 -ALD, 2 FBPase, 2 -PGI, 2 -HK, RK, AK, APRT, ANase, -PRPPsyn, ADK) |
| 9. | Riboneogenesis from glycerol and 3PGA  2 3PGA + 2 Gly 🡪 2 Rib  (2 -PGK, 2 -GAPDH, 2 -GK, 2 -GDH, 2 -ALD, 2 FBPase, 2 -PGI, 2 G6PDH, 2 PGL, 2 6PGDH, 2 PPI, 3 RK, AK, APRT, ANase, -PRPPsyn, ADK, 4 NADPHu) |
