# Supplementary material for: Handling Uncertainty in Dynamic Models: The Pentose Phosphate Pathway in Trypanosoma brucei
Source: PLoS Comput Biol. 2013 Dec 5;9(12):e1003371. doi: 10.1371/journal.pcbi.1003371 (PMC3854711; doi:10.1371/journal.pcbi.1003371)

**Text S1. Uncertainty modeling & distribution of parameter values**

The sampling methodology used is the same as described in [1]. Each parameter is sampled independently while respecting thermodynamic constraints, usually from a log-normal distribution using experimentally observed standard deviations. Thus, the distributions used to sample the parameters reﬂect our uncertainty about the value of each parameter. In comparison to [1], the time limit allowed for a given model to reach steady-state (from the initial values based on the steady-state of the ﬁxed-parameter model) is increased to 1000 minutes (instead of 300) to account for the larger size of the models. This limit is further extended to 10,000,000 for the simulations of the inhibition of 6-phosphogluconate dehydrogenase (Figures 5, S6–7) to allow most models to reach steady-state. Despite this large time limit, a small proportion of the models still are unable to reach steady-state before the time limit due to the accumulation of some metabolites (Figures S8–9).

Except for time series simulations, the results presented in the main manuscript are the steady state values of only those models that were able to reach steady state within 1000 minutes. For the glycolysis part of the models, the parameters and their uncertainties are the same as in [1] with the exception of the following parameters:

- The V_max_ of the phosphoglycerate mutase is still unknown. The accumulation of 3-phosphoglycerate described in [1] when this V_max_ is sampled in the lower range of its values is still occurring after addition of the pentose phosphate pathway. This issue is not modified by the addition of the pentose phosphate pathway in any of the topologies described in the main text. However, in order to limit the percentage of models that fail to reach steady state before 1000 minutes, we limited the sampling of the V_max_ of the phosphoglycerate mutase so that 95 % of the models have values in the range [202.5; 250] with a median of 225 nmol · min^–1^ · mg protein^–1^ (distribution is log normal [1]).
- The V_max_ of the pyruvate transporter is set using the median value and standard deviation calculated from [2] as described in [1]. Similarly, as for the phosphoglycerate mutase, the accumulation of pyruvate observed when the V_max_ of the pyruvate transporter is in the lower range of its values is not modified by the addition of the pentose phosphate pathway.

**New parameters:** The parameters of the newly added reactions are sampled using the same rules as used for the glycolysis parameters. The median values are set as the fixed parameter values described in the main article unless specified otherwise below. The standard deviations are set as described in the sources of the fixed-parameter values, when these mention a standard deviation or standard error of mean or using the mean standard error of the other measured parameters as described in [1].

**Exceptions:** The following parameter values remains fixed unless mentioned otherwise:

- The parameters of the black-box reactions of NADPH utilization in the cytosol and the glycosome.
- The parameters of the ATP:ADP antiporter.
- The parameter *k_TOX_* of trypanothione oxidation.

**V_max_ of enzymes present in both the cytosol and the glycosome:** The total activity is sampled as described above. The cytosolic fraction is then computed from this total activity and the fixed percentage of activity as described in the main article, the rest of the activity is the glycosomal activity. This percentage of cytosolic activity is fixed except for the cytosolic hexokinase activity, which is sampled from a uniform distribution between 1 and 10 percent, as its value has an important impact on some of the results as described in the main article.

**Glucose-6-phosphate phosphatase V_max_:** This value is unknown. It is sampled from a uniform distribution between 1 and 40 nmol · min^–1^ · mg protein^–1^ (this latter value is in the range of the measured total phosphatase activity reported in [3]).

**Ribokinase V_max_:** This value is unknown. As the equilibrium constant of ribokinase favors the reverse direction more easily, we sampled the reverse V_max_ with a median of 1000 nmol · min^–1^ · mg protein^–1^ and 97.5 % of the value sampled lower than 4000 nmol · min^–1^ · mg protein^–1^ (log normal distribution).

**Equilibrium constants**: In addition to the equilibrium constants of the pentose phosphate pathway reactions, equilibrium constants were introduced for pyruvate kinase, hexokinase and phosphofructokinase as described in the methods subsection of the main manuscript. All the new equilibrium constants are sampled using the fixed-parameter value as a median and the relative standard error of the other equilibrium constants, except for glucose-6-phosphate dehydrogenase for which the value used is the median of the values published in [4,5] corrected for pH (median equilibrium constant = 7.0), and the corresponding standard error.

**Distributions**

Distribution of parameters and steady-state ﬂuxes and concentrations in the sampled model based on model C (see Figure 1 of the main article). Additionally, 1000 parameter sets of the models including the transport of glucose only are available as Dataset S2, and 250 parameter sets of the models including the transport of both glucose and fructose are available as Dataset S3. Most parameters are sampled from a log normal distributions of mean and standard deviations based on experimental sources, some parameters are calculated from other sampled parameters for thermodynamical consistency. Detailed descriptions can be found in the section above and in [1]. The vertical black lines indicate the value in the fixed- parameter model. The units of the x-axis are as follows: K_m_, K_i_ and K_s_: mM; V_max_: nmol · min^–1^ · mg protein^–1^; others: unitless.

1. Achcar F, Kerkhoven EJ, The SilicoTryp Consortium, Bakker BM, Barrett MP, et al. (2012) Dynamic modelling under uncertainty: the case of Trypanosoma brucei energy metabolism. PLoS Comput Biol 8: e1002352. doi:10.1371/journal.pcbi.1002352.

2. Vanderheyden N, Wong J, Docampo R (2000) A pyruvate-proton symport and an H+-ATPase regulate the intracellular pH of Trypanosoma brucei at different stages of its life cycle. Biochem J 346 Pt 1: 53–62.

3. McLaughlin J (1986) The association of distinct acid phosphatases with the flagella pocket and surface membrane fractions obtained from bloodstream forms of Trypanosoma rhodesiense. Mol Cell Biochem 70: 177–184.

4. Wurster B, Hess B (1970) Kinetic analysis of the glucosephosphate isomerase-glucose-6-phosphate dehydrogenase system from yeast in vitro. Hoppe-Seyler’s Z Physiol Chem 351: 1537–1544.

5. Wurster B, Hess B (1973) Anomeric specificity and anomerase activity of glucosephosphate isomerase (EC 5.3.1.9) From baker’s yeast. Hoppe-Seyler’s Z Physiol Chem 354: 407–420.


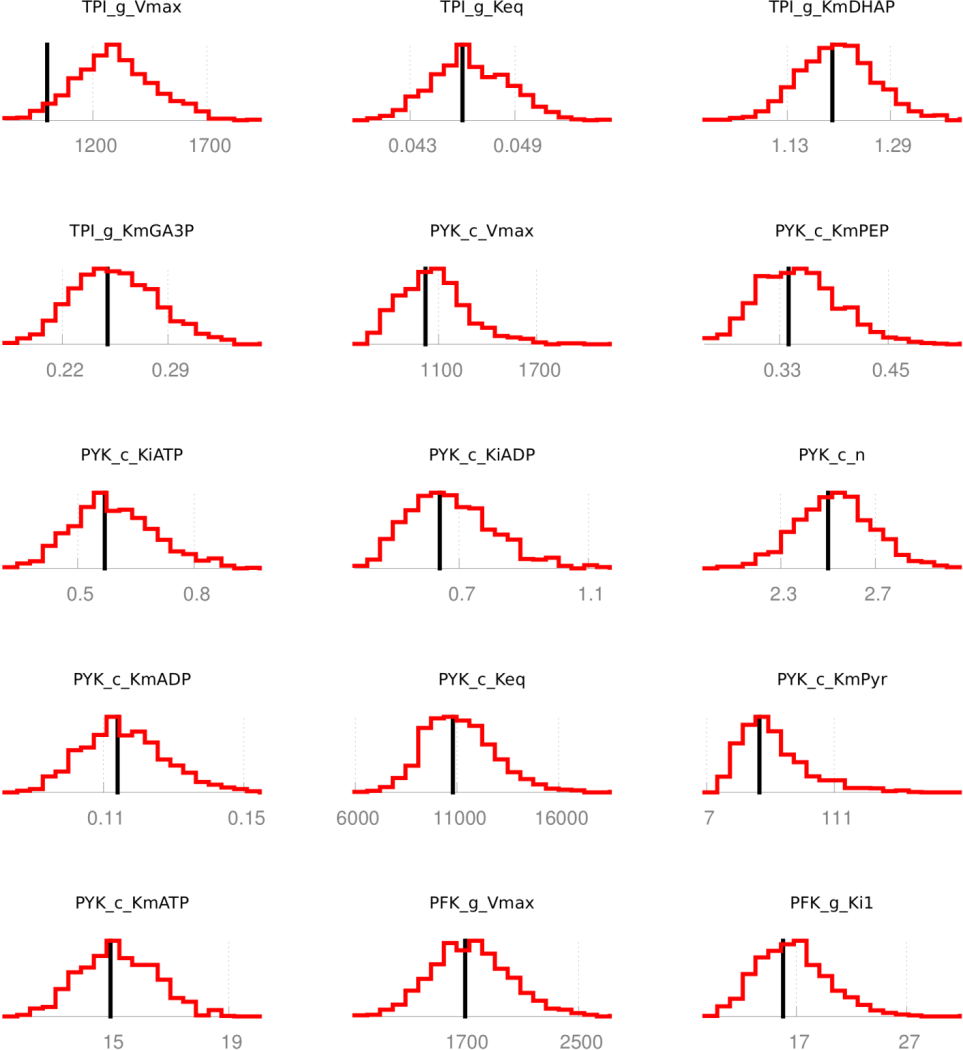


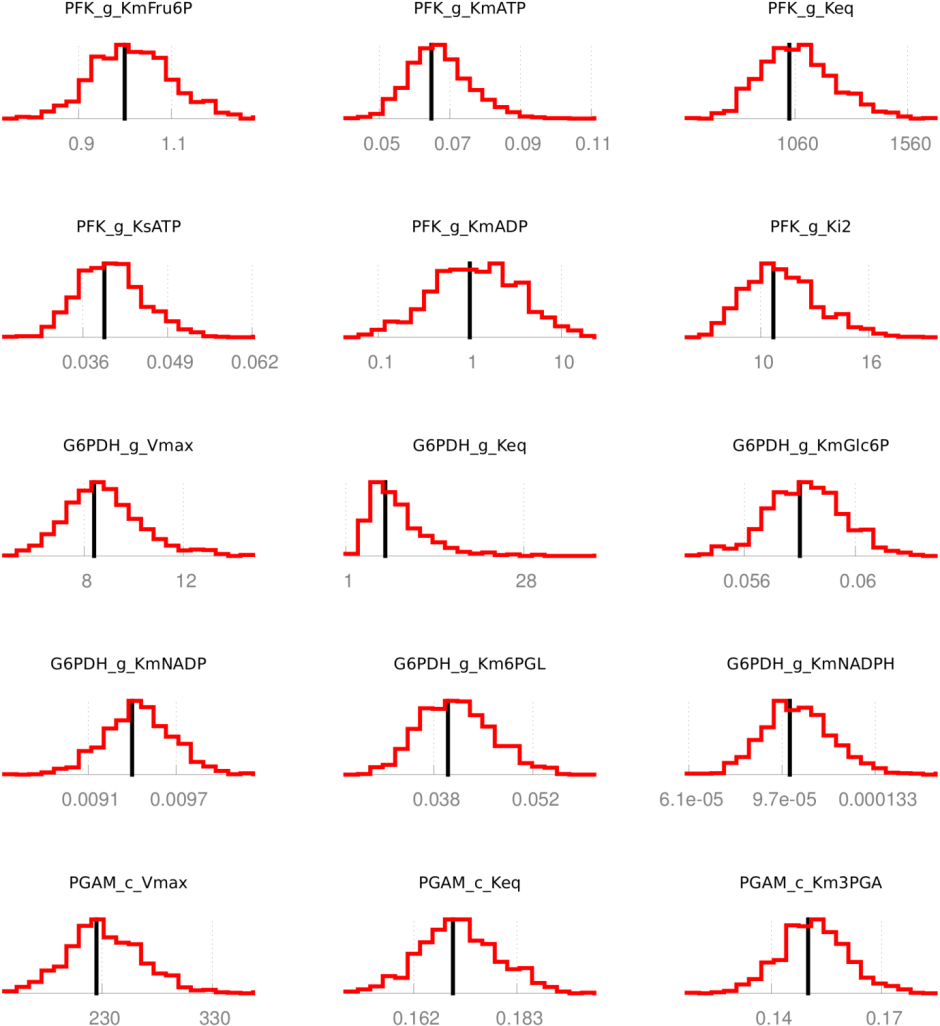

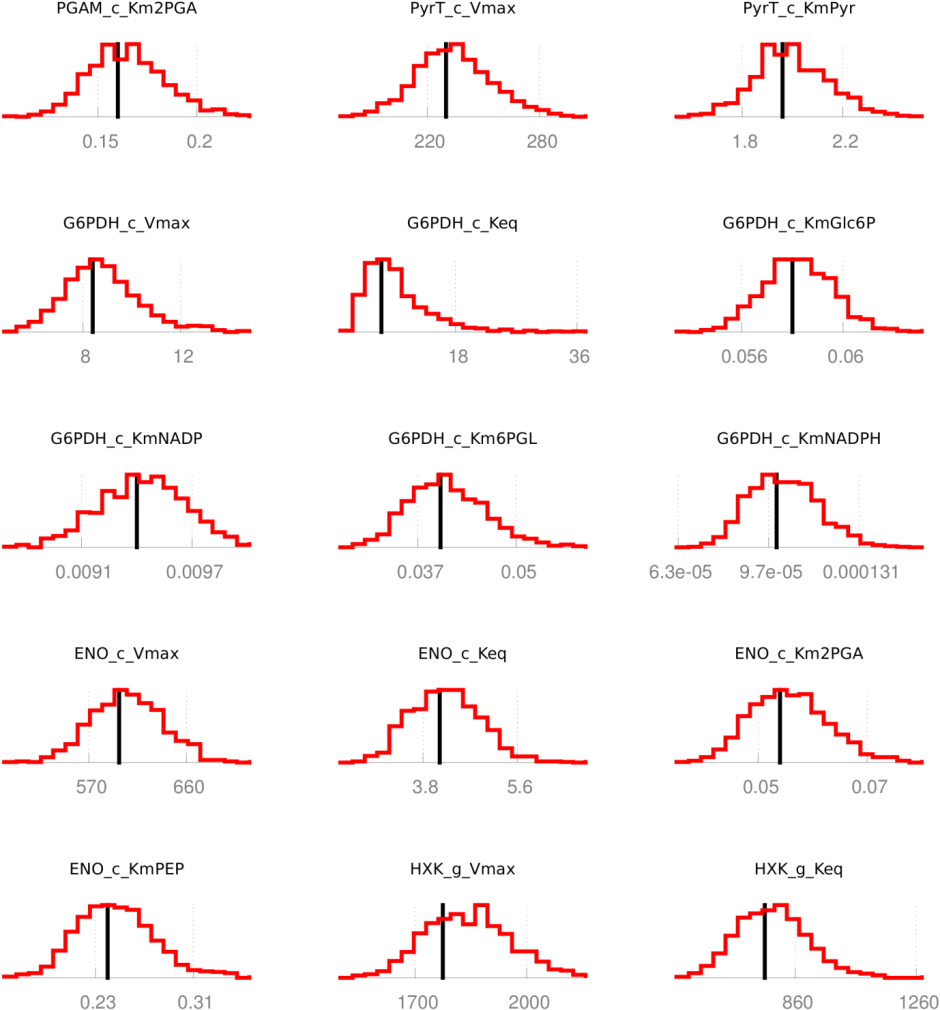

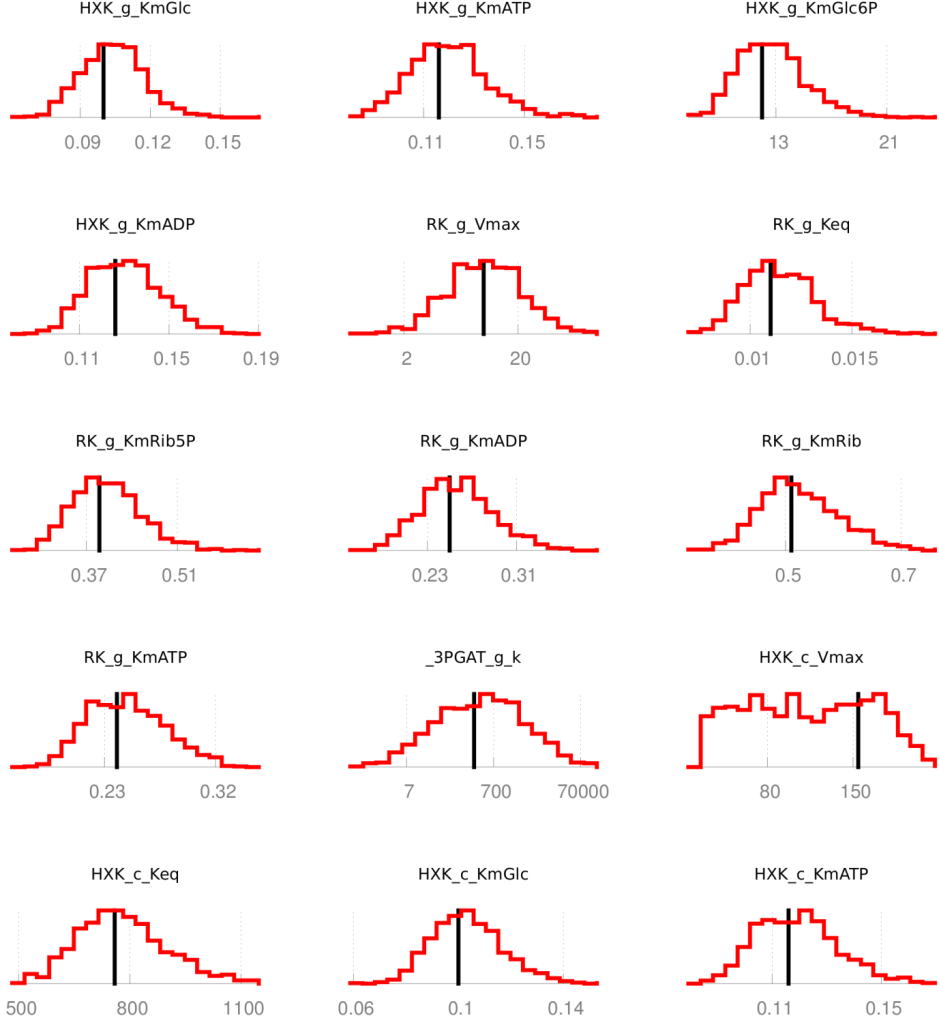

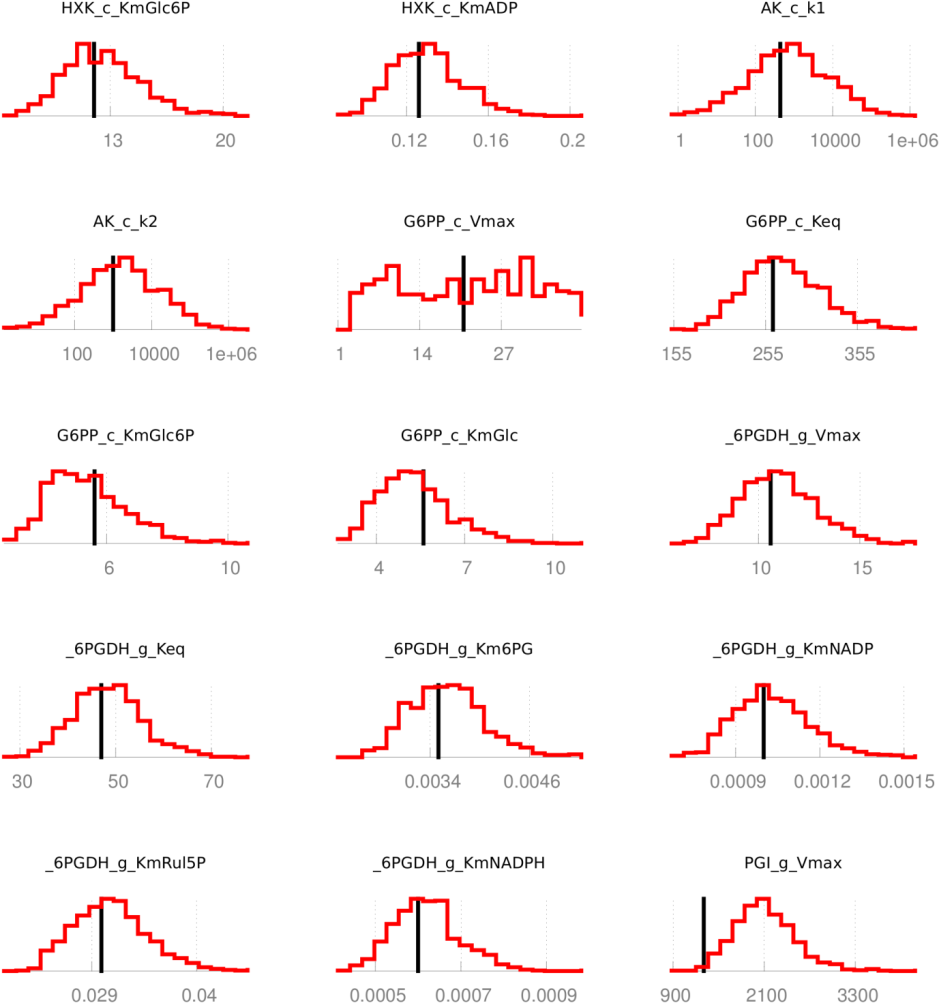

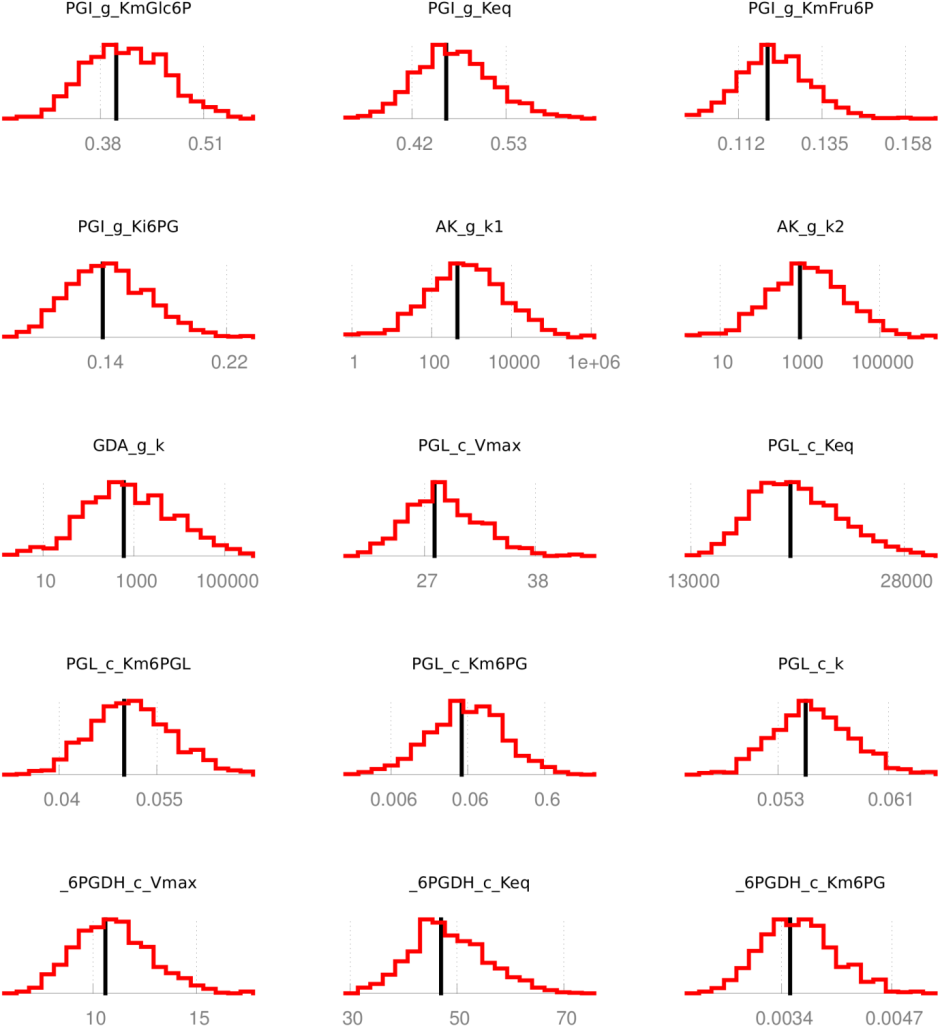

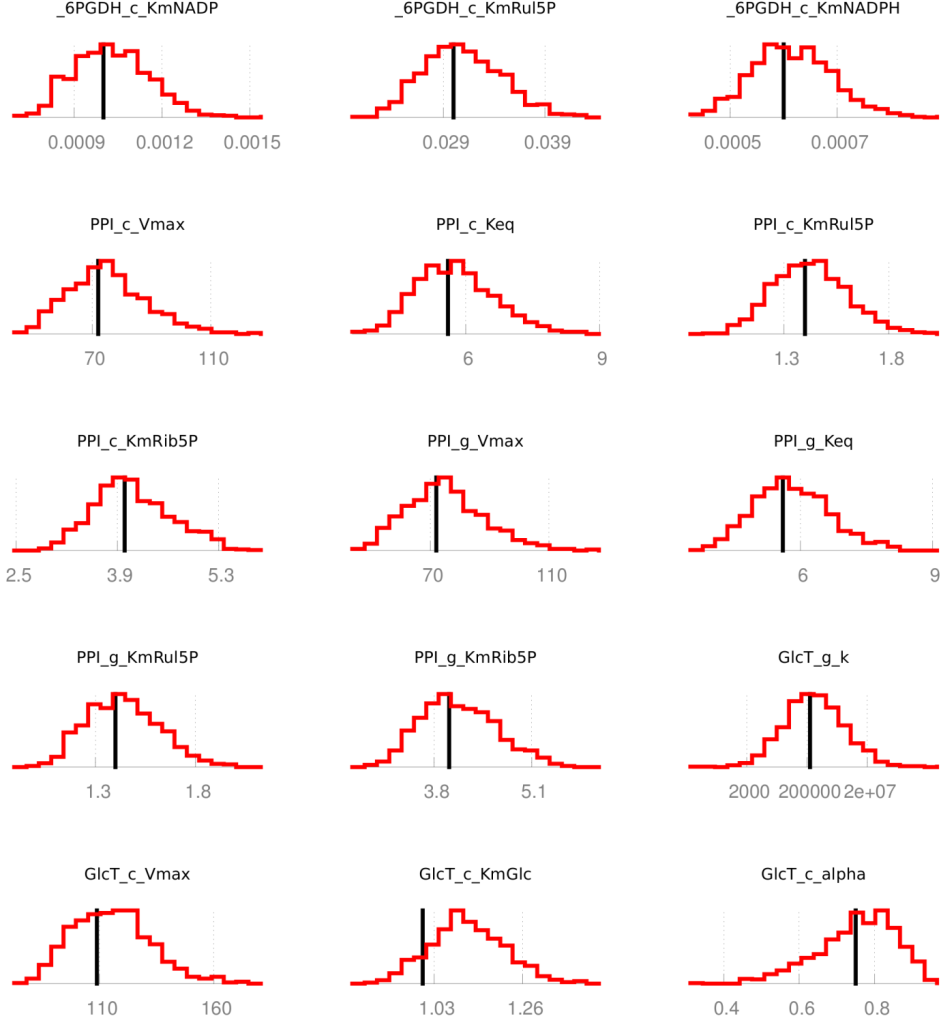

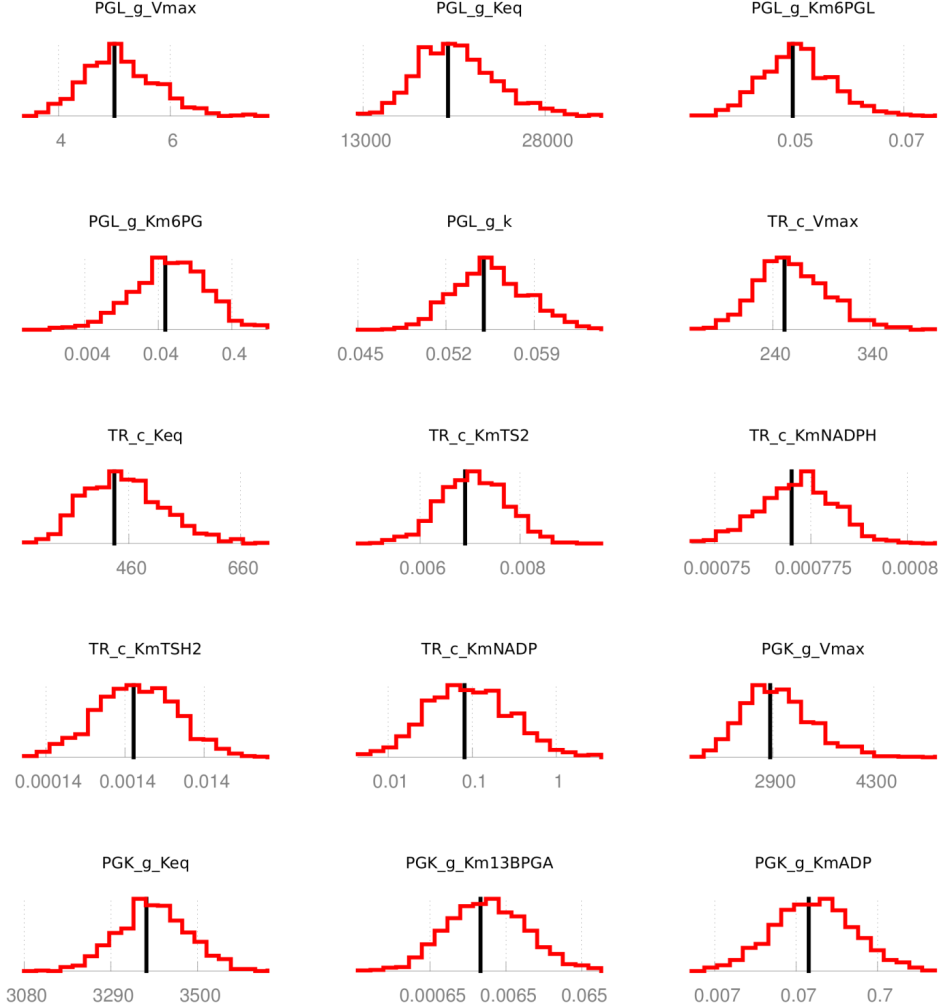

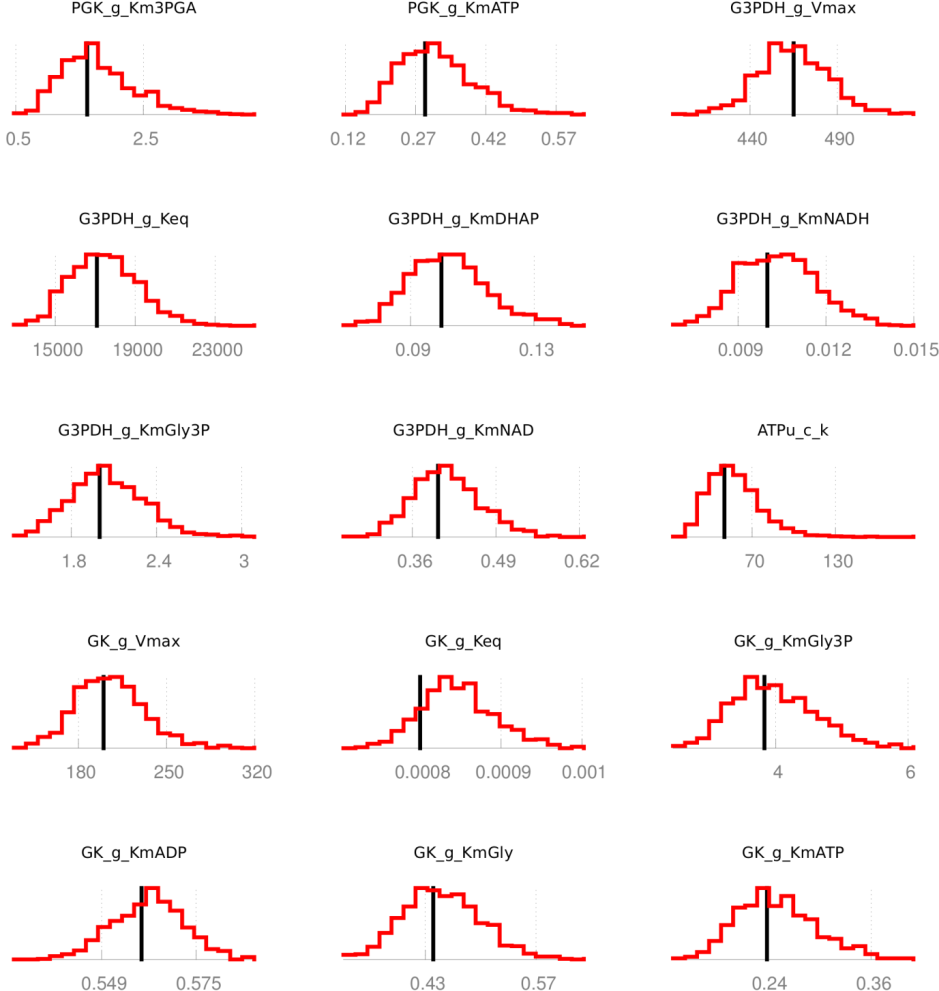

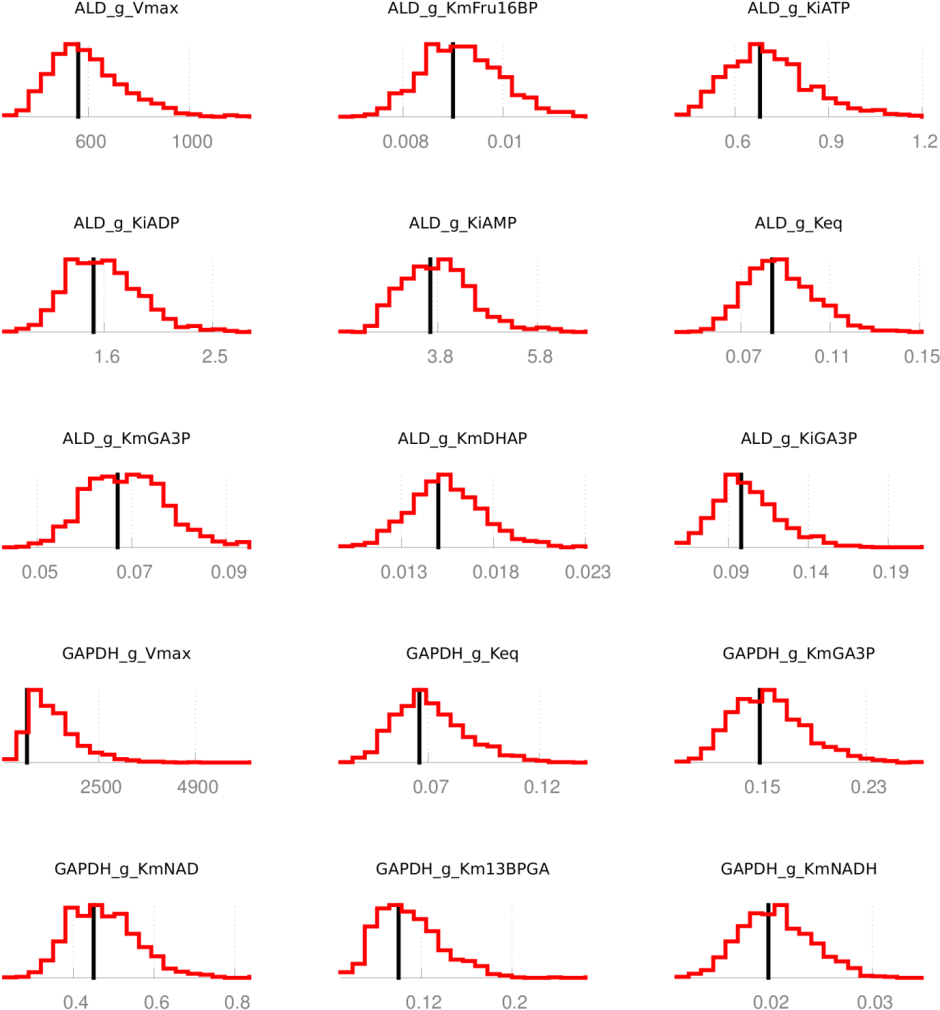

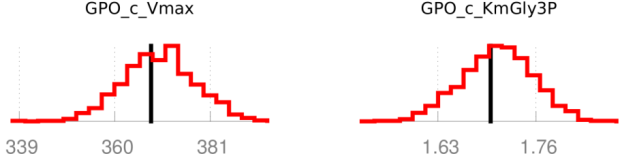

Supplement: Text S1 — Uncertainty modeling and distribution of parameter values. Detailed description of the methodology for uncertainty modeling and distributions defined for sampling of random parameter values. (DOCX) [file pcbi.1003371.s017.docx]
